# Correction of Anemia in Chronic Kidney Disease With *Angelica sinensis* Polysaccharide via Restoring EPO Production and Improving Iron Availability

**DOI:** 10.3389/fphar.2018.00803

**Published:** 2018-07-31

**Authors:** Kaiping Wang, Jun Wu, Jingya Xu, Saisai Gu, Qiang Li, Peng Cao, Mingming Li, Yu Zhang, Fang Zeng

**Affiliations:** ^1^Hubei Key Laboratory of Natural Medicinal Chemistry and Resource Evaluation, Tongji Medical College of Pharmacy, Huazhong University of Science and Technology, Wuhan, China; ^2^Department of Pharmacy, Union Hospital, Tongji Medical College, Huazhong University of Science and Technology, Wuhan, China

**Keywords:** anemia, chronic kidney disease, erythropoietin, iron, polysaccharide

## Abstract

Given the limited efficacy and potential disadvantages of erythropoiesis-stimulating agents (ESAs) in treating anemia of chronic kidney disease (CKD), the development of better alternative therapies has become a priority. The primary purpose of this study is to investigate the effects of *Angelica sinensis* polysaccharide (ASP) and its underlying mechanism in the treatment of renal anemia. In the present study, we found that ASP could enhance hypoxic induction of EPO in Hep3B cells, with a mechanism that involved the stabilization of HIF-2α protein. In parallel, ASP rescued the inhibition of EPO, induced by proinflammatory factor TNF-α through blocking GATA2 and NF-κB activation. In a rat model of adenine-induced anemia of CKD, oral administration of ASP corrected anemia and alleviated renal damage and inflammation. By increasing the accumulation of HIF-2α protein and reducing the expression of NF-κB and GATA2 as well as pro-inflammatory cytokines, ASP stimulated both renal and hepatic EPO production, and resulted in an elevation of serum EPO. The restoration of EPO production and EPOR mRNA expression with ASP treatment activated EPOR downstream JAK2/STAT5 and PI3K/Akt signaling, induced their target genes, such as Bcl-xL, Fam132b and Tfrc, and increased Bcl-2/Bax ratio in bone marrow-derived mononuclear cells of CKD rats. Furthermore, we found that ASP suppressed hepatic hepcidin expression, mobilized iron from spleen and liver and increased serum iron. These findings demonstrate that ASP elicits anti-anemic action by restoring EPO production and improving iron availability in the setting of CKD in rats.

## Introduction

Anemia is a frequent complication in many patients suffered from chronic kidney disease (CKD) associated with reduced health-related quality of life, progression of renal dysfunction, and increased cardiovascular disease, hospitalizations and mortality (Li et al., 2005; Soni et al., 2010). Relative deficiency of EPO is the predominant cause of anemia in CKD. EPO, the primary driver of erythropoiesis, is the hormone essential for maintaining the survival, proliferation and differentiation of erythroid progenitor cells in the bone marrow, acting by binding the EPO receptor (EPOR) homodimer on the cell surface of erythroblasts (Cheung and Miller, 2001). The expected compensatory response to anemia is a heightened rate of erythropoiesis by an exponentially increased EPO production in kidneys. However, this response is disturbed and causes anemia in most patients with substantially impaired renal function. EPO is primarily produced by renal EPO-producing cells (REPs) in a hypoxia-inducible manner via the activation of hypoxia-inducible transcription factors (HIFs) (Suzuki and Yamamoto, 2016). Mechanisms demonstrated to account for impaired EPO production in CKD are alterations in the function of REPs and perturbations of the hypoxia-sensing system in the kidney (Souma et al., 2013, 2015). Additionally, inflammatory cytokines, such as TNF-α and IL-1β, have been shown to inhibit EPO production through activating GATA-2 and NF-κB (Frede et al., 1997; La Ferla et al., 2002; Souma et al., 2013).

More recently, it has become apparent that disordered iron homeostasis is another crucial factor contributing to renal anemia (Panwar and Gutierrez, 2016). Many CKD patients have functional iron deficiency or iron blockade within cells of the reticuloendothelial system (RES), characterized by low circulating iron in the setting of normal iron stores (Weiss and Goodnough, 2005). Recent work has elucidated that hepcidin excess may account for this iron retention (Eleftheriadis et al., 2009; Babitt and Lin, 2010). Hepcidin, the central regulator of systemic iron homeostasis, restricts the release of recycled iron in macrophages and stored iron in hepatocytes into plasma, resulting in inadequate iron supply for erythropoiesis (Sebastiani et al., 2016). In CKD patients, hepcidin levels have been found to be highly elevated, presumably due to induction by inflammation and reduced renal clearance (Ashby et al., 2009; Zaritsky et al., 2009; Babitt and Lin, 2010).

Erythropoiesis-stimulating agents (ESAs; epoetin or darbepoetin alfa) and intravenous iron supplementation have become the mainstay of anemia therapy in CKD patients. Administration of ESAs is generally effective, but can also result in adverse effects, like exacerbation of hypertension, increased risk of cardiovascular events, thrombophilia, stroke and death, and clotting of the dialysis lines (Macdougall, 2009). And approximately 10–20% of patients with end-stage kidney disease are found to be resistant to ESAs and require large doses of the agent, which is associated with an increased mortality (Levin and Rocco, 2006). Thus, there is an urgent need for safer and more efficient alternative strategies to treat renal anemia.

The root of *Angelica sinensis* (Oliv.) Diels (*A. sinensis*), commonly known as dong quai, dang gui or “female ginseng,” has been used for thousands of years in traditional Chinese, Korean, and Japanese medicine. Merck introduced the herb to the Western world in 1899 under the trade name Eumenol, a product designed to ease menstrual pain. Previous clinical evidence showed that regular supplementation of *A. sinensis* improved anemia in a hemodialysis patient who was resistant to recombinant human EPO (rhEPO) therapy (Bradley et al., 1999). In a recent clinical study of renal anemia, a combined treatment with Danggui Buxue Decoction, consisting mainly of *A. sinensis*, Radix astragali and conventional western medicine (CNW), indicated that Danggui Buxue Decoction could improve the therapeutic efficacy of CNW without adverse events (Zhao et al., 2017). However, the underlying mechanism of *A. sinensis* in treating renal anemia is not clear. *Angelica sinensis* polysaccharide (ASP), a water-soluble polysaccharide consisting of glucuronic acid, glucose, arabinose, and galactose, is one of the main active ingredients isolated from *A. sinensis*. In our previous studies, ASP suppressed hepcidin expression and ameliorated iron deficiency anemia (Zhang et al., 2014). Other reports indicated that ASP exerted hematopoietic, anti-inflammatory, and immunomodulatory effects (Yang et al., 2006; Liu et al., 2010; Lee et al., 2012; Wang et al., 2016).

Based on these observations, we hypothesize that ASP ameliorates anemia of CKD by stimulating EPO production, suppressing hepcidin, and attenuating inflammation. To test this hypothesis, we first investigated the effects of ASP toward hypoxia-inducible EPO production and EPO repression by inflammatory cytokines *in vitro*. We then studied the therapeutic effectiveness of ASP in an adenine-induced rat model of CKD that developed renal damage, anemia, inflammation, impaired EPO production, inactivation of EPO receptor signaling and disordered iron metabolism.

## Materials and Methods

### Reagents and Materials

Adenine, CoCl_2_, cycloheximide (CHx), rat EPO ELISA kit, β-actin, Histone H3.1 and horseradish peroxidase (HRP)-conjugated secondary antibodies against mouse or rabbit were purchased from Sigma (St. Louis, MO, United States). Recombinant human erythropoietin (rhEPO) was provided by Sunshine Pharmaceutical Company (Shenyang, China). The biochemical kits of serum creatinine (Scr) and blood urea nitrogen (BUN) were provided by NanJing JianCheng Bioengineering Institute (Nanjing, China). The EPO-producing hepatoma cell line Hep3B was obtained from American Type Culture Collection (ATCC, Manassas, VA, United States). Fetal bovine serum (FBS) and penicillin-streptomycin were obtained from Gibco (Grand Island, NY, United States). Recombinant human TNF-α and IL-1β were obtained from PeproTech (Rocky Hill, Connecticut, United States). Trizol and Cy3-conjugated goat anti-rabbit IgG were from Invitrogen (Carlsbad, CA, United States), and Ficoll-Paque PREMIUM were from GE Healthcare (Chicago, United States). The All-in-One^TM^ First-Strand cDNA Synthesis kit and All-in-One^TM^ qPCR SYBR Green Mix were obtained from GeneCopoeia (United States). Nuclear/Cytosol Extract kit, protease inhibitors, phosphatase inhibitors and bicinchoninic acid (BCA) protein assay kit were obtained from Beyotime (Shanghai, China). Primary antibodies against HIF-1α(ab179483), HIF-2α(ab179825), GATA2 (ab109241), ferritin (ab109373) and ferroportin (ab78066), were purchased from Abcam (Cambridge, England). Primary antibodies against NF-κB p65 (#8242), p-SMAD1/5/8 (#13820), p-STAT3 (#9145), p-JAK2 (#3776), p-STAT5 (Tyr694) (#4322), p-PI3K (#4228), and p-Akt (#4060) were purchased from Cell Signaling Technology (Danvers, MA, United States). The enhanced chemiluminescence kit was from Thermo scientific (Waltham, MA, United States). Other reagents and chemicals were of analytical grade.

### Preparation and Structure Characterization of *Angelica sinensis* Polysaccharide

The dry roots of *A. sinensis* (Umbelliferae) were obtained from Union Hospital and collected from Minxian (Gansu Province, China) in October 2015. Plant identification was undertaken by Professor Jinlan Ruan (Faculty of Pharmaceutical Science, Tongji Medical College of Huazhong University of Science and Technology, Wuhan, China) in accordance with the identification standard of the Pharmacopoeia of People’s Republic of China. The polysaccharide from *A. sinensis* was isolated and purified as previously described (Zhang et al., 2016). The purity of ASP was approximately 95.6% and molecular weight was 80 kDa. The structure characterization of ASP was determined as previously described (Zhang et al., 2016). ASP was an acidic heteropolysaccharide consisting of glucuronic acid, glucose, arabinose and galactose in ratio of 1.00:1.70:1.85:5.02. The main chains of ASP were composed of (1→3)-linked galactopyranose (Gal*p*), (1→6)-linked Gal*p* and 2-OMe-(1→6)-linked Gal*p*, which had three side chains attached to O-3 of 2-OMe-(1→6)-linked Gal*p*, and terminated with (1→)-linked glucopyranuronic acid (Glc*p*A) and arabinofuranose (Ara*f*). The structure of the repeating unit of ASP was shown in Supplementary Figure S1.

### Cell Culture and Treatment

Hep3B cells were maintained in DMEM medium supplemented with 10% heat-inactivated FBS and 1% penicillin/streptomycin solution at 37°C with 95% air and 5% CO_2_. For hypoxic conditions, the cells were incubated with 50 μM CoCl_2_ for 24 h. In hypoxia experiments, Hep3B cells were incubated with medium or ASP (100 and 200 μg/mL) under hypoxic conditions for 24 h. These ASP concentrations were chosen based on cell viability assays (shown in Supplementary Figure S2) and a pre-test study. For assessing protein stability, cells were incubated in the presence of CoCl_2_ with or without ASP for 24 h and then treated with 25 μg/mL CHx for indicated times. In TNF-α stimulation experiments, Hep3B cells were incubated with 25 ng/mL recombinant human TNF-α in presence or absence of ASP (100 and 200 μg/mL) under normoxia and hypoxia for 24 h.

### Animals and Experimental Protocol

All animal protocols were approved by *the Institutional Animal Care and Use Committee of Tongji Medical College, Huazhong University of Science and Technology* (Wuhan, China) (NO. SCXK2016-0009). The animal care and experimental procedures were carried out in accordance with *the Guidelines of the Institutional Animal Care and Use Committee of Tongji Medical College and the National Institutes of Health Guide for the Care and Use of Laboratory Animals*. Fifty male Sprague-Dawley rats (6-week old, 160–180 g) were purchased from the Center for Experimental Animal Research (Wuhan, China) and kept on a standard rodent diet. The animals had free access to food and water and were housed with a 12 h light-dark cycle and an average temperature of 20°C ± 1°C. They were acclimatized for 1 week before being used for the experiment.

For the adenine model, rats (*n* = 40) were fed a 0.75% adenine supplemented diet for 4 weeks followed by a regular diet for 4 weeks (see **Figure 1**). Alternatively, rats (*n* = 10) were given a regular diet (150.4 mg Fe/kg, the Center for Experimental Animal Research, Wuhan, China) throughout all 8 weeks (control group). The adenine-fed rats were randomly divided into four groups of ten rats each: one vehicle-treated adenine group (adenine group), one rhEPO-treated group (EPO group), and two ASP intervention groups (0.5 and 1 g/kg per day, referred as LASP group and HASP group). These ASP dosages were chosen based on a previous study (Zhang et al., 2012, 2014). RhEPO treatment was given weekly, starting from week 2 at a dose of 500 U/kg body weight by intraperitoneal (i.p.) injection. ASP was dissolved in distilled water and administered orally by metallic gavage needle, starting from week 2. Rats of control group and adenine group were orally administrated of distilled water (1 mL per day). Tail vein phlebotomy (0.5–1 mL) were performed every 1 or 2 weeks. Blood was collected into ethylene diamine tetra-acetic acid (EDTA)-containing vacuum tubes and eppendorf tubes without anticoagulant, respectively. After 4 h at room temperature, blood samples in eppendorf tubes were centrifuged at 5000 rpm/min for 10 min. Supernatant serum was stored at −20°C until analysis. Rats were anesthetized by an injection of 3% sodium pentobarbital (150 mg/kg) at week 8, then, the rats were euthanized by cervical dislocation. The kidneys, livers and spleens were harvested for iron content, protein and RNA extraction, or immunohistology.

**FIGURE 1:**
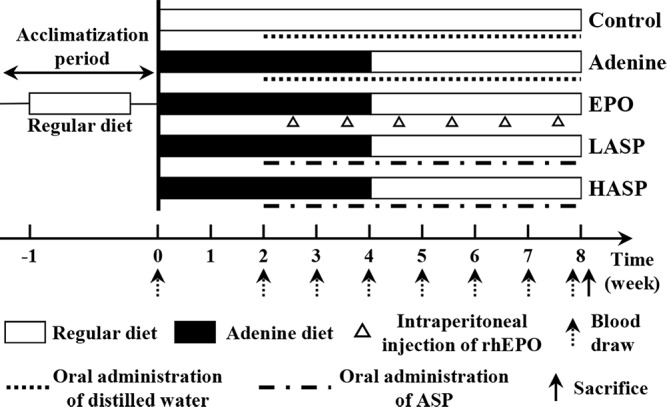
Schematic of ASP treatment strategy in an adenine-induced anemia of CKD model. After a 1-week acclimation period, male Sprague-Dawley rats were given either a regular diet (open bars) for 8 weeks (control) or a 0.75% adenine supplemented diet (black bars) for 4 weeks followed by a regular diet (open bars) for another 4 weeks. The adenine diet group was further treated with distilled water alone (adenine) or ASP at two dosages [0.5 (LASP) or 1.0 (HASP) g/kg] daily or recombinant human erythropoietin (EPO) once a week, while the control group was treated with distilled water alone, starting at week 2. Tail-vein blood draws were performed at weeks 0, 2, 3, 4, 5, 6, 7, and 8 for all groups. Animals were sacrificed at week 8.

### Hematological, Iron and Biochemical Analysis

Blood in EDTA-containing vacuum tubes was analyzed by an automatic blood counter (MEK-6318K, NIHON KOHDEN, Tokyo, Japan) for the determination of Hb, red blood cell (RBC) counts and white blood cell (WBC) counts. Non-heme iron in spleen and liver and serum iron were determined with a flame atomic-absorption spectrometer (Model AA-240 FS, VARIAN Co., Palo Alto, CA, United States). Scr and BUN were used as renal function markers and determined using biochemical kits according to the manufacturer’s protocol. Serum EPO levels were measured using commercially available rat EPO ELISA kit.

### Tissue Preparation for Histologic Analysis

Kidney tissues were fixed in 4% paraformaldehyde solution followed by decalcification process for 2 weeks. After decalcification, the samples were processed, embedded in paraffin and sectioned. Afterwards, 4 μm thick sections for routine histopathological studies were stained with hematoxylin and eosin (H&E). Photomicrographs were taken with an optical microscope equipped with a camera (Nikon Eclipse Ci, Nikon Corporation, Tokyo, Japan).

### Isolation of Bone Marrow-Derived Mononuclear Cells (BM-MNCs)

The femoral bones were aseptically removed from rats and cleaned of adherent soft tissues. And the bones were washed three times for 5 min each wash in DMEM medium supplemented with 1% penicillin/streptomycin solution. Following the removal of the distal portions of femur, the marrow cavity was flushed out using 10 mL of culture medium (DMEM with 10% heat inactivated fetal bovine serum) expelled from a 10 mL syringe through a 25 gauge needle to obtain bone marrow cell suspensions. Cells were suspended in 1 × PBS and centrifuged on Ficoll-Paque PREMIUM (density 1.084 g/cm^3^) by density gradient centrifugation at 2000 r/min for 30 min at 20°C to obtain the mononuclear cells. Cell number and viability were assessed after staining with trypan blue. The BM-MNCs were harvested for mRNA and protein analysis.

### Immunofluorescence Staining

Hep3B cells were fixed with 4% formaldehyde and treated with ice-cold methanol for 10 min at −20°C. After blocking in PBS with 5% BSA and 0.3% Triton X-100 for 1 h at room temperature, the cells were incubated with NF-κB p65 (1:400) diluted in PBS with 1% BSA and 0.3% Triton X-100 at 4°C overnight. Cy3-conjugated goat anti-rabbit IgG was used as the secondary antibody and was diluted in PBS with 0.1% Tween 20 and incubated for 1 h in the dark, followed by staining with DAPI for another 10 min. The slides were observed by a fluorescence microscope (Nikon E400, Nikon Corporation, Tokyo, Japan).

### Quantitative Real-Time PCR

Total RNA was extracted from Hep3B cells, kidneys, livers, spleens or BM-MNCs using Trizol according to the manufacturer’s protocol. Complementary DNA was synthesized using the All-in-One^TM^ First-Strand cDNA Synthesis kit. Quantitative reverse transcription polymerase chain reaction (qRT-PCR) was carried out in an ABI 7900 real-time PCR system (Illumina, San Diego, CA, United States) for 40 cycles using the All-in-One^TM^ qPCR SYBR Green Mix. Gene expression levels were calculated based on the threshold cycle (Ct) values and the efficiency of each primer set. The results were expressed as the means of fold changes in target gene expression relative to β-actin (2^−ΔΔCt^) ± SEM, *n* = 3. The specific primer pairs used for PCR are listed in Supplementary Table S1.

### Western Blotting

Nuclear extracts were prepared from TNF-α-treated Hep3B cells using a commercially Nuclear/Cytosol Extract kit and were used for western blotting analysis of GATA2 and NF-κB p65 expression. Total protein extracts were prepared from Hep3B cells or nitrogen frozen rat kidneys, livers, spleens and BM-MNCs in RIPA lysis buffer (50 mM Tris-HCI at pH 7.4, 150 mM NaCl, 1% Triton X-100, 1% sodium deoxycholate, 0.1% SDS) containing PMSF, protease inhibitors and phosphatase inhibitors. Protein content was determined by BCA assay, and 80 μg of total protein were separated by 10% SDS-PAGE and transferred to nitrocellulose membrane. Membranes were immunoblotted with antibodies against HIF-1α, HIF-2α, NF-κB p65, GATA2, p-JAK2, p-STAT5, p-PI3K, p-Akt, p-SMAD1/5/8, p-STAT3, ferritin, and ferroportin. β-actin or Histone H3.1 were used as a loading control. To detect signals, horseradish peroxidase-conjugated secondary antibodies against rabbit or mouse were used with enhanced chemiluminescence staining agents. Protein levels were quantified by densitometry using Image J programs (version 1.48v, National Institutes of Health, United States).

### Statistical Analysis

Data were presented as means ± standard error of mean (SEM). Comparisons between multiple groups in this study were performed by one-way analysis of variance (ANOVA) with either LSD (assuming equal variances) or Dunnett’s T3 (not assuming equal variances) for *post hoc* analysis. A corrected *P*-value less than 0.05 was regarded as statistically significant. All data were analyzed with SPSS version 19.0 software (SPSS, Chicago, IL, United States). All Graphs were performed with GraphPad Prism 5.02 (GraphPad Software, United States).

## Results

### ASP Increases the Cellular Levels of HIF-1/2α by Blocking Protein Degradation and Stimulates EPO Production During Hypoxia in Hep3B Cells

To verify whether ASP could stimulate hypoxia-inducible EPO production, we first examined the effects of ASP on HIFs and EPO expression *in vitro*. The human hepatoma cell lines Hep3B, which are so far the most prevalently used models for studying hypoxia-regulation of EPO expression *in vitro*, were utilized in our present study (Chun et al., 2001; Nakano et al., 2004; Warnecke et al., 2004). Hep3B cells were treated with 50 μM CoCl_2_ for 24 h to mimic the hypoxic conditions. Western blot was performed on total proteins harvested from Hep3B cells treated with medium or ASP for 24 h under hypoxic conditions. In Hep3B cells, HIF-1α protein expression was strongly induced under hypoxic conditions, but HIF-2α protein expression was not affected (**Figures 2A,B**). After the treatment with ASP, the protein expression of both HIF-1α and HIF-2α was dose-dependently increased (**Figures 2A,B**). Next, we analyzed the EPO mRNA levels. Hypoxia significantly induced EPO mRNA expression, and ASP treatment (at the dose of 200 μg/mL) resulted in a twofold increase of EPO mRNA level over the medium-treated group under hypoxic conditions (**Figure 2C**).

**FIGURE 2:**
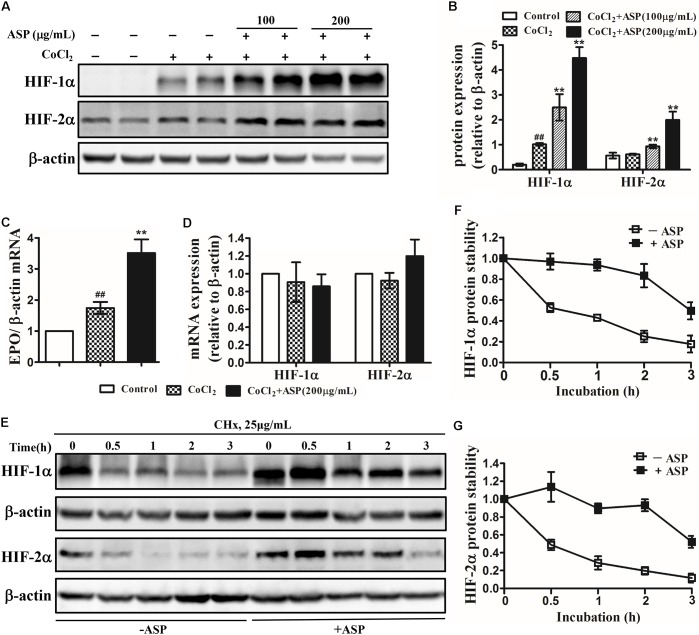
Effects of ASP on HIF-1α and HIF-2α expression, EPO mRNA expression and HIF-1α and HIF-2α protein stability in hypoxic Hep3B cells. Analyses of **(A,B)** HIF-1α and HIF-2α protein expression, **(C)** EPO mRNA expression, and **(D)** HIF-1α and HIF-2α mRNA expression. **(E–G)** Hep3B cells were treated with or without 200 μg/mL ASP in the presence of 50 μM CoCl_2_ for 24 h. Then, the cells were harvested at 0, 0.5, 1, 2, and 3 h following the addition of 25 μg/ml cycloheximide (CHx) to determine HIF-1α and HIF-2α protein levels by western blot analysis. **(F)** HIF-1α and **(G)** HIF-2α expression was corrected for β-actin and normalized such that the band intensity in lane 0 h was set at one. One representative blot out of three independent experiments is shown β-actin served as the loading control. Values are means ± SEM from three independent experiments. ^##^*P* < 0.01 compared with the control group; ^∗∗^*P* < 0.01 compared with the CoCl_2_ group. *Open diamond*, without ASP treatment; *closed diamond*, with ASP treatment.

To further ascertain whether ASP affects the mRNA expression or protein stability of HIF-1/2α, we first analyzed the mRNA expression of HIF-1α and HIF-2α. Results demonstrated that neither HIF-1α nor HIF-2α mRNA expression was augmented by ASP treatment under hypoxic conditions (**Figure 2D**). Next, we measured protein half-life after blocking *de novo* protein synthesis with cycloheximide. As shown in **Figures 2E–G**, the half-life of HIF-1α and HIF-2α were both prolonged to 3 h in the presence of ASP, whereas that in the absence of ASP was 0.5 h. This results suggested that ASP further stabilized HIF-1/2α protein under hypoxic conditions.

### ASP Reverses the Inhibition of EPO Production Induced by TNF-α Through Blocking GATA2 and NF-κB Activation in Hep3B Cells Under Normoxia and Hypoxia

Given that TNF-α and IL-1β are the major inflammatory cytokines inhibiting EPO production, we then used recombinant human TNF-α and IL-1β for EPO suppression in Hep3B cells. As shown in **Figure 3A**, the nuclear GATA2 expression induced by TNF-α was dose-dependently suppressed by ASP treatment. Similarly, ASP at the dose of 200 μg/mL significantly decreased the nuclear translocation of NF-κB (**Figure 3B**). Meanwhile, EPO mRNA expression was reduced by 54%, which was ameliorated by ASP in a dose-dependent manner. In parallel, ASP blocked IL-1β-induced GATA2 and NF-κB, and abrogated the suppression of EPO mRNA by IL-1β (Supplementary Figures S3A–C).

**FIGURE 3:**
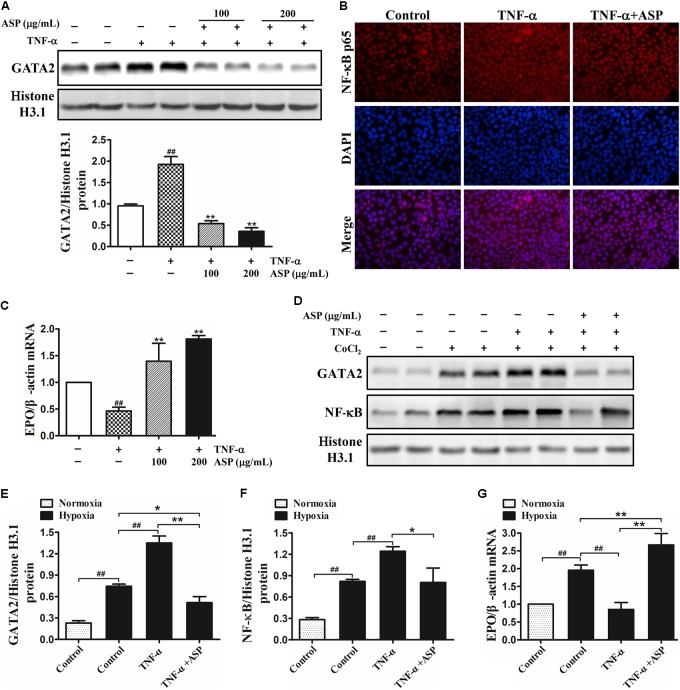
Effects of ASP on TNF-α-induced activation of GATA2 and NF-κB and on inhibition of EPO induced by TNF-α in Hep3B cells under normoxia and hypoxia. **(A)** Expression of nuclear GATA2 in TNF-α-induced Hep3B cells. **(B)** Immunofluorescence analysis of NF-κB nuclear translocation by ASP treatment (200 μg/mL). Original magnification, 400×. **(C)** EPO mRNA expression in TNF-α-induced Hep3B cells. **(D–F)** Nuclear GATA2 and NF-κB expression of TNF-α-induced Hep3B cells cultured in the presence of CoCl_2_ for 24 h. **(G)** EPO expression of TNF-α-induced Hep3B cells under hypoxia. One representative blot out of three independent experiments is shown, Histone H3.1 served as nuclear loading control. Values are means ± SEM from three independent experiments. ^##^*P* < 0.01 compared with the control group; ^∗^*P* < 0.05, ^∗∗^*P* < 0.01 compared with the TNF-α group.

Then, Hep3B cells were treated with 25 ng/mL TNF-α in the presence or absence of ASP and exposed to 50 μM CoCl_2_ for 24 h. As illustrated in **Figure 3D**, hypoxia induced GATA2 and NF-κB expression relative to normoxia, and the addition of TNF-α further enhanced GATA2 and NF-κB expression. By contrast, ASP completely abolished the induction by TNF-α and partially suppressed the induction by hypoxia compared with normoxia (**Figure 3D**). Consistently, TNF-α stimulation diminished hypoxia-induced EPO mRNA, and ASP reversed the inhibition of EPO by TNF-α under hypoxia and further augmented hypoxia-induced EPO (**Figure 3E**).

### ASP Attenuates Adenine-Induced Chronic Renal Failure in Rats

As presented in **Figures 4A,B**, adenine-fed rats demonstrated a significant increase in BUN and Scr that peaked at week 4. Following the feeding of regular diet, both BUN and Scr levels started falling, but remained a significant increase from week 5 to the week 8 compared to controls, which indicated persistent kidney disease. Strikingly, ASP treatment significantly decreased Scr and BUN levels in rats as early as 2 weeks after treatment initiation compared with animals receiving vehicle control (**Figures 4A,B**). Afterward, the BUN levels fell rapidly to levels similar to those of the untreated CKD rats, and the Scr levels continuously declined and reached its nadir at week 8 (**Figures 4A,B**). Kidneys were removed from rats at week 8 and weighed, and then processed for routine H&E staining for histological study. The kidneys were significantly enlarged in the adenine-fed rats, while ASP treatment dose-dependently reduced kidney indices (**Figure 4C**). As shown in **Figure 4D**, the adenine-induced CKD rats exhibited significant glomerular and tubulointerstitial lesions, and marked deposition of crystalline bodies were observed in the renal tubules and interstitium (**Figure 4D**). Moreover, renal tubular atrophy, and inflammatory cells infiltration and fibrosis in the interstitium were also found. These findings were compatible with symptoms of chronic renal failure. Treatment with ASP attenuated renal histopathological damage (**Figure 4D**).

**FIGURE 4:**
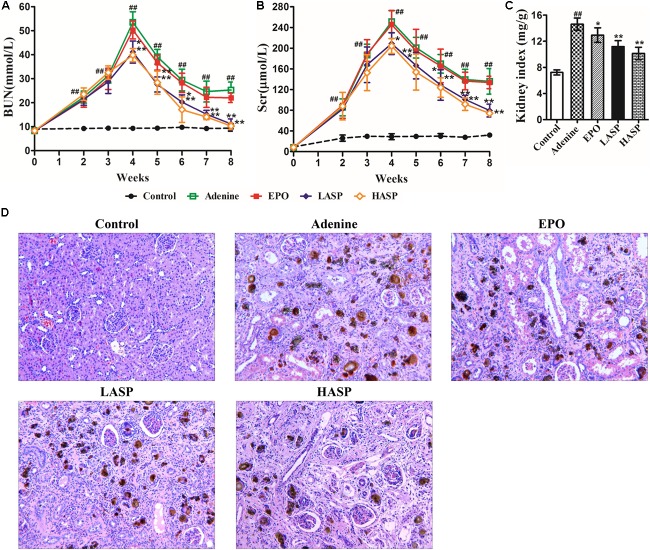
Renal function markers and histopathological examination of kidney in adenine-induced rat model. Evaluation of **(A)** BUN and **(B)** Scr throughout the experimental protocol. **(C)** Kidney weight was normalized to animal body weight. **(D)** H&E staining in kidneys. Original magnification, 200×. Data in **(A–C)** are expressed as means ± SEM (*n* = 8–10 per group and time point). ^##^*P* < 0.01 compared with the control group; ^∗^*P* < 0.05, ^∗∗^*P* < 0.01 compared with the adenine group. BUN, blood urea nitrogen; Scr, serum creatinine.

### ASP Ameliorates Anemia and Inflammation in an Adenine-Induced CKD Model

Adenine-induced CKD rats were followed by weekly complete blood counts to observe the development and resolution of anemia. The CKD rats developed a prolonged anemia associated with kidney disease compared with control rats. There was a dramatic decrease of Hb and RBC at week 4 that reached a nadir at week 6 (**Figures 5A,B**; mean adenine Hb 95 g/L vs. control Hb 148 g/L; mean adenine RBC 4.83 × 10^12^/L vs. control RBC 7.78 × 10^12^/L) and persisted to week 8 with Hb 106 g/L and RBC 5.27 × 10^12^/L. We treated rats starting at week 2 of the adenine diet. Compared with untreated animals, ASP-treated rats developed less severe anemia, exhibited markedly elevated Hb and RBC levels as early as at week 6 and recovered completely at week 8 (**Figures 5A,B**). In parallel, the rhEPO-treated rats did not develop anemia, showing the normal levels of Hb and RBC during the whole experimental period (**Figures 5A,B**). Additionally, we observed a significant increase in spleen index, manifesting splenic extramedullary hematopoiesis. And ASP treatment markedly reduced the spleen index compared with adenine group, while rhEPO treatment further increased it (**Figure 5C**).

**FIGURE 5:**
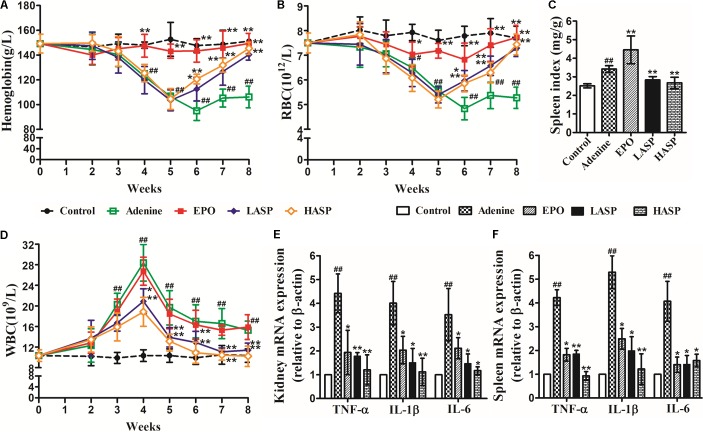
Effects of ASP treatment on hemoglobin, RBC, spleen index, WBC as well as renal and splenic pro-inflammatory cytokine expression in a rat model of CKD **(A)** Hemoglobin, **(B)** RBC numbers, **(C)** spleen index and **(D)** WBC numbers. Eight to ten rats for each group and time point were analyzed. mRNA expression of TNF-α, IL-1β, and IL-6 in **(E)** kidney and **(F)** spleen (*n* = 3). Results are reported as means ± SEM. ^#^*P* < 0.05, ^##^*P* < 0.01 compared with the control group; ^∗^*P* < 0.05, ^∗∗^*P* < 0.01 compared with the adenine group. CKD, chronic kidney disease; RBC, red blood cell; WBC, white blood cell.

The adenine-induced CKD rats also developed chronic inflammation. Feeding of adenine diet with rats resulted in an evident elevation in WBCs as early as 3 weeks after the start of feeding, with an eventual peak at week 4 (**Figure 5D**). Following the feeding of regular diet, WBC levels started to fall, but remained a significant increase from week 5 to the week 8 compared to controls (**Figure 5D**). Rats receiving ASP treatment greatly improved their leukocytosis. After 2 weeks of ASP treatment (at week 4), the rats showed a prominent reduction in the number of WBCs compared with untreated CKD animals. Then, the WBC numbers continued to decline and returned to baseline by week 7. Whereas weekly injection of rhEPO had no effect on leukocytosis in adenine-fed rats. We further assessed the inflammatory response by measuring pro-inflammatory cytokines expression in kidney and spleen at the end of the experimental period. Quantitative RT-PCR analysis demonstrated that the mRNA expression of TNF-α, IL-1β, and IL-6 in kidney were notably increased by 4.4 times, 4 times and 3.5 times, respectively (**Figure 5E**). We found that ASP treatment dose-dependently suppressed TNF-α, IL-1β, and IL-6 mRNA expression, and this effect was paralleled by corresponding changes in the spleen (**Figures 5E,F**).

### ASP Restores EPO Production by Augmenting HIF Signaling and Reducing NF-κB and GATA2 Expression in CKD Rats

Subsequently, we evaluated the effect of ASP on EPO expression in CKD rats. Indeed, serum EPO concentration in CKD rats was not different from that in control rats (**Figure 6A**). However, EPO mRNA expression in kidneys was notably reduced by 47% (**Figure 6B**). Further analysis of EPO expression in liver revealed that hepatic EPO mRNA expression was significantly increased (average a 1.8-fold increase relative to control) (**Figure 6C**). In comparison with that in untreated CKD rats, treatment with ASP dose-dependently enhanced renal EPO mRNA, which was paralleled by corresponding changes in the liver (**Figures 6B,C**). In particular, administration of ASP largely improved serum EPO levels in rats fed with adenine chow (**Figure 6A**).

**FIGURE 6:**
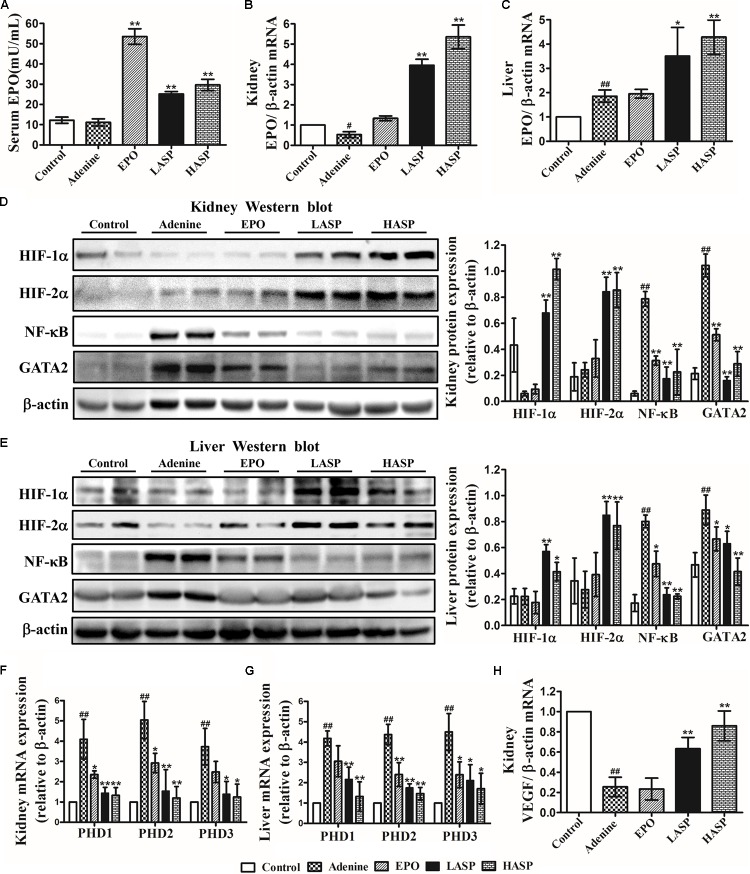
Effects of ASP treatment on EPO production, NF-κB and GATA2 expression and HIF signaling in an adenine-induced CKD model. **(A)** Serum EPO levels. Ten rats for each group were analyzed. **(B)** Kidney and **(C)** liver EPO mRNA analysis (*n* = 3). Protein expression of HIF-1α and HIF-2α as well as NF-κB and GATA2 in **(D)** kidney and **(E)** liver were analyzed by western blot in three independent experiments. mRNA expression of PHD1, PHD2, and PHD3 in **(F)** kidney and **(G)** liver as well as **(H)** VEGF in kidney (*n* = 3). One representative blot out of three independent experiments is shown, β-actin served as the loading control. Results are expressed as means ± SEM. ^#^*P* < 0.05, ^##^*P* < 0.01 compared with the control group; ^∗^*P* < 0.05, ^∗∗^*P* < 0.01 compared with the adenine group.

To study the mechanisms underlying the induction of EPO by ASP, we analyzed HIF signaling as well as the inhibitors of EPO gene, NF-κB and GATA2. As the activity of HIFs is mainly regulated by HIF prolyl hydroxylases (or prolyl hydroxylase domain proteins PHDs: PHD1, 2, and 3), the effect of ASP on the PHDs was also investigated. As presented in **Figures 6D–G**, the level of accumulation of HIF-1/2α in adenine-fed rats was not significantly different from that in control rats, and adenine-fed rats showed a markedly elevation of PHD1/2/3 mRNA in kidney and liver. And we revealed that administration of ASP with CKD rats resulted in a significant reduction of PHD1/2/3 mRNA and a robust accumulation of HIF-1/2α protein both in kidney and liver (**Figures 6D–G**). Besides, NF-κB and GATA2 expression both in kidney and liver were strongly induced in CKD rats, but repressed by ASP treatment (**Figures 6D,E**). Further analysis of VEGFα another HIF target gene, indicated that ASP attenuated the suppression of VEGFα and the expression of VEGFα still remained below that of controls (**Figure 6H**).

### ASP Treatment Enhances the Expression EPOR and EPOR Signaling Cascades in BM-MNCs

To further determine the effects of EPO restoration with ASP treatment on downstream EPOR signaling systems, we analyzed EPOR expression, the downstream JAK2/STAT5 and PI3K/AKT signaling and their target gene in BM-MNCs. Results indicated that EPOR mRNA levels were extremely low in the adenine-induced CKD rats, whereas this reduction was partially antagonized by ASP treatment in a dose-dependent fashion (**Figure 7A**). In addition, EPO treatment of rats also significantly enhanced EPOR mRNA, which, however, remained lower than that in ASP-treated rats (**Figure 7A**). When performing western blot analysis in the BM-MNCs, we found that p-JAK2 and p-STAT5 levels as well as p-PI3K and p-Akt levels were strongly suppressed in CKD rats as compared to control rats (**Figures 7B,C**). Both ASP and EPO treatment resulted in a trend toward increased expression of p-JAK2 and p-STAT5 as well as p-PI3K and p-Akt (**Figures 7B,C**).

**FIGURE 7:**
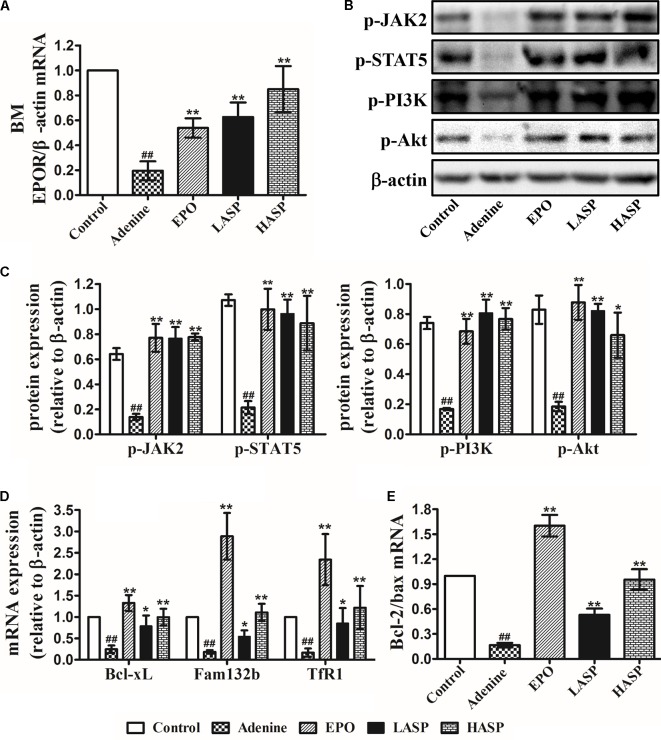
Effects of ASP treatment on EPOR expression and EPOR downstream signaling cascades in BM-MNCs of rats. **(A)** EPOR mRNA levels. **(B)** Representative immunoblots of p-JAK2 and p-STAT5 as well as p-PI3K and p-Akt. **(C)** Protein expression in **(B)** were quantified by densitometry. mRNA expression of **(D)** Bcl-xL, fam132b (ERFE) and TfR1 (TfRc) as well as **(E)** Bcl-2 and Bax. One representative blot out of three independent experiments is shown, β-actin served as the loading control. Data are means ± SEM from three independent experiments. ^#^*P* < 0.05, ^##^*P* < 0.01 versus the control group. ^∗^*P* < 0.05, ^∗∗^*P* < 0.01 versus the adenine group. EPOR, EPO receptor; BM-MNCs, bone marrow-derived mononuclear cells; BM, bone marrow; ERFE, erythroferrone.

Based on the observations made thus far, we questioned whether the restoration of EPO/EPOR signaling may inhibit apoptosis in BM-MNCs. We next examined the Bcl-xL gene, known as a target of STAT5 and known to strongly suppress apoptosis in erythroid progenitors (Socolovsky et al., 1999). Results demonstrated that the Bcl-xL mRNA expression was dramatically decreased in BM-MNCs derived from adenine-induced CKD rats, whereas ASP treatment attenuated the decrease of Bcl-xL, which was paralleled by EPO therapy (**Figure 7D**). In line with the changes of Bcl-xL, we found a dramatic decrease of Bcl-2/Bax ratio in the BM-MNCs of adenine-fed rats, and this reduction was prevented by ASP and EPO treatment (**Figure 7E**).

We proceeded to analyze the mRNA expression of TfRc, also known as TfR1 or CD71 expressed on the erythroblast surface at very high levels for the intake and utilization of iron in erythroblasts, and erythroferrone (ERFE, Fam132b gene) that is secreted by erythroblasts immediately after EPO stimulation and suppresses the expression of hepcidin (Kautz et al., 2014; Kim and Nemeth, 2015). Results showed that the expression of TfR1 and Fam132b was extremely lower in CKD rats as compared with that in rats fed with regular chow (**Figure 7D**). ASP treatment led to a significant rise of TfR1 and Fam132b mRNA, which became more prominent by EPO therapy (**Figure 7D**).

### ASP Inhibits Hepcidin, Mobilizes Spleen and Liver Iron Stores and Improves Hypoferremia in CKD Rats

We next assessed the effects of ASP on iron metabolism. Adenine-induced rats exhibited a significant reduction of serum iron and had higher spleen iron and liver iron contents than control rats (**Figures 8A,B**), an indication of reticuloendothelial cell iron blockade. After 6 weeks of ASP treatment, serum iron levels were markedly elevated, and the tissue iron stores in spleen and liver were dramatically reduced (**Figures 8A,B**). Inflammatory cytokines, mainly IL-6, stimulates hepcidin by promoting phosphorylation of STAT3, which acts together with BMP/SMAD pathways (Nemeth et al., 2004a; Besson-Fournier et al., 2012). We, therefore, investigated the effect of ASP on hepcidin as well as p-STAT3 and p-SMAD1/5/8. Liver hepcidin mRNA expression was significantly increased by 3.6 times in adenine-fed rats, which was paralleled by increased phosphorylation of STAT3 and SMAD1/5/8 in livers of adenine-fed rats compared with control animals (**Figures 8C–E**). As shown in **Figure 8C**, both two doses of ASP as well as EPO significantly decreased liver hepcidin mRNA expression as compared to that in untreated CKD rats. Especially, hepcidin mRNA expression in high-dose of ASP-treated animals was returned to baseline (**Figure 8C**). In parallel, liver p-STAT3 and p-SMAD1/5/8 were tremendously lowered in ASP-treated rats compared with adenine-fed rats receiving vehicle administration (**Figures 8D,E**).

**FIGURE 8:**
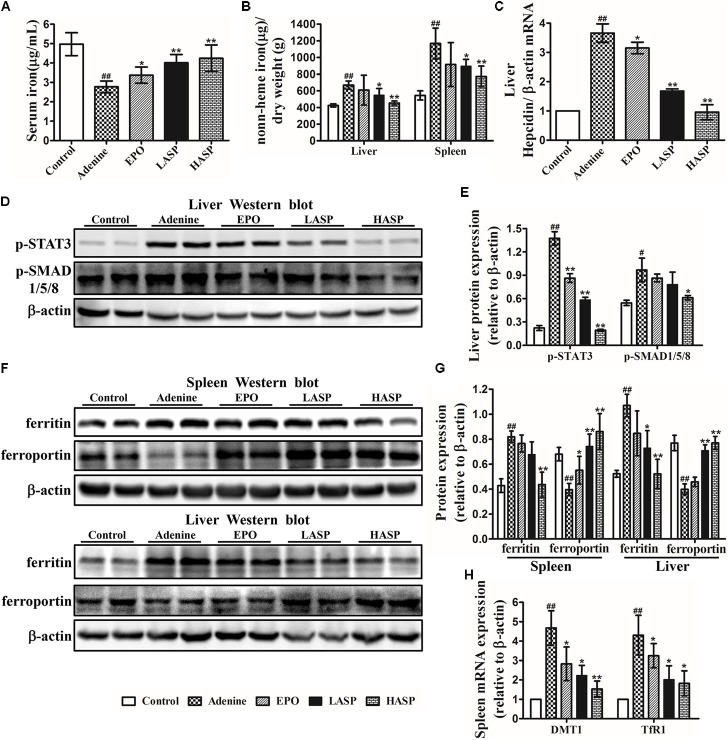
Effects of ASP on iron metabolism in rats with renal anemia. **(A)** Serum iron as well as **(B)** spleen and liver iron were analyzed with a flame atomic-absorption spectrometer (*n* = 8–10 per group). **(C)** Hepcidin (Hamp) relative to β-actin mRNA by quantitative real-time RT-PCR (*n* = 3). Protein expression of **(D,E)** p-STAT3 and p-SMAD1/5/8 in liver as well as **(F,G)** ferritin and ferroportin expression in spleen and liver were analyzed by western blot in three independent experiments. **(H)** DMTI and TfR1 mRNA levels by qRT-PCR were measured in the spleen of rats (*n* = 3). One representative blot out of three independent experiments is shown, β-actin served as the loading control. Results are reported as means ± SEM. ^#^*P* < 0.05, ^##^*P* < 0.01 compared with the control group; ^∗^*P* < 0.05, ^∗∗^*P* < 0.01 compared with the adenine group.

Next, we determined whether the ASP-mediated hepcidin inhibition was responsible for the iron transfer from spleen and liver to plasma. When performing western blot analysis of the spleen and liver, we found that ferroportin expression was suppressed in adenine-fed rats, but ferritin expression was strongly induced (**Figures 8F,G**). Treatment with ASP enhanced ferroportin expression in these two organs, while the ferritin expression was declined by ASP treatment. Moreover, we observed increased mRNA expression of TfR1 and DMT1 in spleen, which was repressed by ASP treatment (**Figure 8H**).

## Discussion

The root of *A. sinensis* is a herbal medicine for the treatment of anemia, menstrual disorders, dysmenorrhea and inflammation in China and other Asian countries. ASP is one of the main bioactive components extracted from *A. sinensis*. Recently, natural polysaccharides have drawn much attention for their low toxicity and costs as well as beneficial therapeutic properties. The intestinal absorption of polysaccharide is a major concern for its use *in vivo* because of its high molecular weight. Our previous study indicated that ASP could be absorbed after oral administration through endocytosis process and circulated into blood (Wang et al., 2017). The present study reports that oral administration of ASP effectively corrects anemia by stimulating renal and hepatic EPO production and increasing iron availability in an adenine-induced rat model of renal failure (**Figure 9**). ASP is an efficient regulator that enhances hypoxia-induced EPO production by stabilizing HIF-2α protein and rescues the inhibition of EPO production induced by inflammation *in vitro* and *in vivo* (**Figures 2**, **3**, **6**). The restoration of EPO production as well as EPOR expression activates EPOR downstream signaling cascades (**Figure 7**), thereby resulting in the stimulation of erythropoiesis and correction of anemia. And by inhibiting hepcidin expression and reducing inflammation, ASP ameliorates functional iron deficiency and increases iron availability (**Figure 8**), which restores iron supply for hemoglobin synthesis and possibly improves the responsiveness and efficacy of EPO.

**FIGURE 9:**
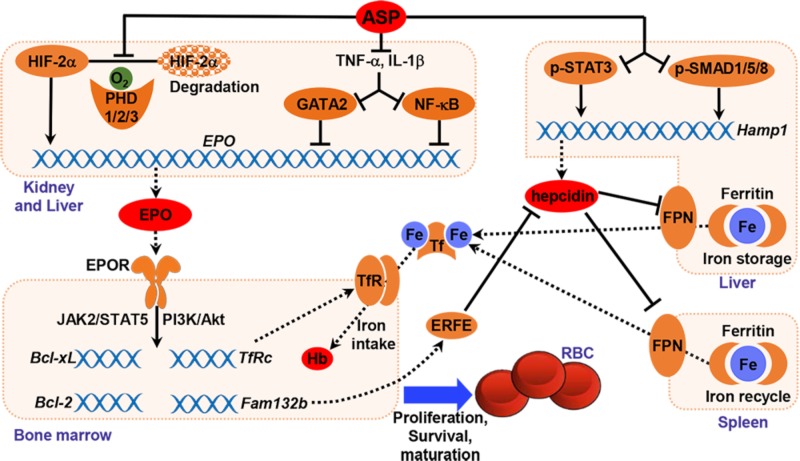
Graphic representation of the underlying mechanism of ASP in treating renal anemia.

Red blood cell production is tightly controlled by EPO to maintain tissue oxygen homoeostasis. Systemic EPO concentration is dynamically regulated by the presence of hypoxia or anemia. Serum EPO levels are inversely related to hemoglobin (Hb) levels in non-renal anemic patients. For instance, serum EPO levels in severely anemic patients are increased by 10–100 times, even up to 1000-fold (Erslev, 1991; Artunc and Risler, 2007). However, the EPO levels in renal anemic patients remain generally normal or slightly increased, considered inappropriately low relative to the degree of anemia (Babitt and Lin, 2012). Consistently, we demonstrated that the serum EPO levels in CKD rats remained normal (**Figure 6A**), despite that Hb concentrations were dropped by 45.0 g/L at the end of experiments (**Figure 5A**), showing a relative deficiency of EPO. Remarkably, ASP treatment in rat CKD models enhanced EPO production both in kidney and liver, thereby increasing the serum EPO concentration. The restoration of EPO production by ASP could be ascribed to stabilization of HIF-2α and rescue of inflammatory inhibition.

In kidney and liver, EPO is stringently regulated at the gene transcriptional level, and HIFs are the major activators of EPO gene expression. HIFs are composed of α and β subunits and the α subunit, of which there are 2 main forms, HIF-1α and HIF-2α, is oxygen sensitive. Under normoxic conditions, HIF-α proteins are hydroxylated by PHDs (PHD1, PHD2, and PHD3), resulting in their proteasomal degradation through von Hippel Lindau protein (pVHL)-mediated ubiquitination (Haase, 2013). In a hypoxic state, the activity of PHDs is inhibited and HIF-α proteins escape degradation. Accumulated HIF-α proteins translocate to nucleus and form a functional heterodimer with the HIF-β subunit, which binds to the hypoxia-responsive element in target genes including EPO, and induces their expression (Haase, 2013). Several lines of evidence suggest that the diseased kidneys in CKD are hypoxic (Eckardt et al., 2005; Nangaku, 2006). However, the injured kidneys of CKD rats did not show a robust accumulation of HIF-1/2α proteins compared with the kidneys of control rats and PHD1/2/3 mRNA expression was significantly increased, suggesting disrupted hypoxia-responsive pathways. Our data support previous studies that demonstrated hypoxic response is impaired or insufficient in injured kidneys, resulting in insufficient HIF-mediated cellular responses (Fahling et al., 2013; Souma et al., 2013, 2016). Preliminary evidence came from both clinical development of PHD enzyme inhibitors and experimental research suggests that augmenting HIF signaling to stimulate EPO production is an attractive strategy for the treatment of anemia of CKD (Souma et al., 2016; Locatelli et al., 2017). The *in vitro* experiment indicated that ASP was an efficient regulator that increased HIF-1/2α protein stability and further enhanced hypoxia-induced EPO production (**Figure 2**). When administrated to CKD rats, ASP reduced PHD1/2/3 mRNA expression and produced a robust increase of HIF-1/2α proteins in kidney and liver, thereby increasing both renal and hepatic EPO production (**Figure 6**). Although the level of hepatic EPO production is weak and insufficient to compensate for anemia in CKD, stimulating hepatic EPO by pharmacologic HIF stabilization is sufficient to improve anemia in models of anemia caused by either renal insufficiency or chronic inflammation (Kapitsinou et al., 2010; Querbes et al., 2012; Yamazaki et al., 2013). In this regard, the increase of serum EPO comes from not only renal EPO restoration, but hepatic EPO stimulation. Further evidence indicates that inflammatory signals activate PHDs and result in degradation of HIFs and impairment of EPO production under pathologic hypoxic conditions (Souma et al., 2015, 2016). Accordingly, the potential effect of ASP on the enhancement of HIF-1/2α proteins in CKD rats could be attributed to the direct blockage of protein degradation we describe in the cells, and probably also to the anti-inflammatory activity of ASP, which would prevent the activation of PHDs mediated by inflammation.

Hypoxia-inducible transcription factors regulate hundreds of genes and other biological processes. And HIF-2α not HIF-1α is reported to be the primary transcription factor regulating hypoxic induction of EPO both in REPs and hepatocyte (Rankin et al., 2007; Souma et al., 2016). So, combined activation of HIF-1α and HIF-2α may raise a concern regarding undesired complications, such as angiogenesis and worsening kidney disease. In clinical development of PHD enzyme inhibitors, the levels of VEGFα a HIF-1α target gene and a key factor mediated angiogenesis, are monitored to evaluate the safety of PHD enzyme inhibitors. In the present study, we showed that the VEGFα expression was increased by ASP, but didn’t exceed the normal levels (**Figure 6H**). Interestingly, renal function markers, BUN and Scr, and histopathological examination of kidney indicated that ASP treatment resulted in amelioration of renal damage, rather than worsening of kidney disease (**Figure 4**).

The central role of inflammation on EPO production is underscored by the observations that anemic patients without renal failure who have inflammation have relatively decreased levels of serum EPO as compared to levels in similarly anemic patients without inflammation (Baer et al., 1987; Miller et al., 1990). CKD is a chronic inflammatory state. In CKD patients, increased levels of inflammatory markers such as C-reactive protein and the cytokines IL-1β, IL-6, IFN-γ, and TNF-α were observed (Kalantar-Zadeh et al., 2003; Stenvinkel et al., 2005). Thus, the impaired EPO production in CKD patients results not only from insufficient HIF signaling but from suppression of EPO transcription by inflammatory cytokines. Our data revealed that ASP reversed the decrease in EPO induced by TNF-α and IL-1β by blocking NF-κB and GATA2 activation both under normoxia and hypoxia *in vitro* (**Figure 3** and Supplementary Figure S3). In CKD rats, administration of ASP attenuated the induction of NF-κB and GATA2 both in kidney and liver (**Figures 6D,E**), and also reduced the expression of TNF-α and IL-1β (**Figures 5E,F**), which would rescue the inhibition of EPO gene transcription by inflammation and further facilitate EPO restoration.

By specifically binding to EPOR on the erythroid progenitors, EPO promotes the activation of intracellular signaling including JAK2/STAT5 and PI3K/Akt pathways. The activation of intracellular signaling induces the expression of genes related to erythroid maturation, proliferation, survival (e.g., Bcl-xL, Bcl-2, and Bax), and iron metabolism (e.g., TfR1 and Fam132b), which prevents apoptosis of erythroid progenitors and results in the utilization of iron. EPO deficiency may increase apoptosis of erythroid progenitors and lead to decreased reticulocyte production. Previous studies analyzing the endogenous EPO levels in CKD patients with anemia and in patients with chronic heart failure (CHF) indicate that, in addition to relative EPO deficiency, bone marrow response to endogenous EPO is inhibited in these patients (Van der Putten et al., 2008). The sensitivity of erythroid progenitor cells to EPO is related to the expression levels of EPOR on the cell surface. Proinflammatory cytokines have a negative effect on EPOR either by directly reducing the expression of EPOR or by inhibiting the growth of erythroid burst-forming units (BFU-Es) and erythroid colony-forming units (CFU-Es) that express EPOR at very high levels (Taniguchi et al., 1997; Macdougall and Cooper, 2002; Means, 2003). Here, we have demonstrated that adenine-induced CKD rats exhibited decreased EPOR mRNA, which was restored by ASP treatment (**Figure 7A**). Thus, ASP treatment not only increased EPO levels, but might enhance bone marrow response to endogenous EPO by increasing EPOR expression. Analyzing intracellular signaling pathways and target genes indicated that ASP treatment reactivated JAK2/STAT5 and PI3K/Akt signaling pathways, dramatically induced Bcl-xL, TfR1, and Fam132b expression and increased Bcl-2/Bax ratio (**Figures 7B–E**). The protein tyrosine phosphatase SHP-1 and cytokine-inducible SH2-containing protein (CIS) are two critical negative regulator of intracellular signaling, which could attenuate intracellular signaling and cause EPO resistance (Klingmuller et al., 1995; Matsumoto et al., 1997; Sasaki et al., 2000; Van der Putten et al., 2008). Akagi et al. (2004) reported that the levels of SHP-1 are increased in hemodialysis patients who are resistant to EPO therapy. And CIS expression is induced by proinflammatory cytokines. Therefore, the restoration of intracellular signaling cascades could be attributed to increased EPO levels and EPOR expression, and probably is related to attenuation of inhibitory effect mediated by SHP-1 and SOCS. Bcl-2 family members, essential for maintenance cell survival, play key roles in mitochondrial pathway of apoptosis. Bcl-xL and Bcl-2 promote cell survival, whereas Bax facilitate apoptosis (Adams and Cory, 1998). Our results demonstrated that Bcl-xL expression and Bcl-2/Bax ratio in BM-MNCs were significantly increased following ASP treatment, possibly implying reduced apoptosis of erythroid precursors. Upregulation of TfR1 and Fam132b might facilitate iron intake and utilization for hemoglobin synthesis.

Maintenance of an adequate iron supply is essential for the production of hemoglobin. Limited iron availability for erythropoiesis can contribute to both anemia and EPO hyporesponsiveness (Van der Putten et al., 2008). Some CKD patients with iron deficiency show blunted response or even don’t respond to a single EPO injection. Most of the iron need for erythropoiesis comes from the recycling of senescent erythrocytes by macrophages with a small contribution from dietary iron absorption that compensates for the daily losses (Pantopoulos et al., 2012). In the present study, we found a diversion of iron from the circulation into storage site in CKD rats. And oral administration of ASP reduced iron contents in spleen and liver and increased serum iron levels (**Figures 8A,B**), thus restoring iron supply for hemoglobin synthesis. Excessive production of hepcidin limits the efflux of iron into plasma, and increases iron retention in the liver and spleen by binding and inducing degradation of the only known iron exporter, ferroportin (Nemeth et al., 2004b). In CKD rats, hepatic hepcidin expression was high and ferroportin levels in spleen and liver were extremely low (**Figures 8C,F,G**). Moreover, we observed increased expression of the iron storage protein ferritin, DMT1 and TfR1 in spleen and liver (**Figure 8H**), associated with increased intracellular iron storage. These may result from enhanced inflammation and cytokine activities. Indeed, inflammatory cytokines also have a direct effect on iron homeostasis by stimulating DMT1 synthesis in macrophages, inducing ferritin expression, and downregulating ferroportin, by increasing TfR-mediated uptake of transferrin-bound iron into macrophages and by stimulating erythrophagocytosis (Weiss and Goodnough, 2005). Thus, the limited iron availability in CKD rats may be referred to the interaction of hepcidin with ferroportin and cytokine-induced intracellular iron storage. We could demonstrate that ASP treatment suppressed liver hepcidin expression by reducing p-STAT3 and p-SMAD1/5/8 expression (**Figures 8C–E**), and increased ferroportin expression in spleen and liver (**Figures 8F,G**), thereby increasing the efflux of iron from spleen and liver to plasma. Furthermore, the iron storage protein ferritin as well as DMT1 and TfR1 expression were decreased by ASP treatment (**Figures 8F–H**), indicating reduced intracellular iron storage. Accordingly, ASP treatment increased iron availability in CKD rats not only by suppressing hepcidin, but by reducing cytokine-induced intracellular iron storage. Theurl et al. (2014) reported that the high pre-treatment hepcidin level was a limited factor for the efficacy of ESA therapy and proposed a combination therapy of ESA and hepcidin antagonizing agents, which can improved therapeutic efficacy of ESAs. As such, increased iron availability with ASP treatment not only could restore iron supply for erythropoiesis, but might improve the responsiveness and efficacy of EPO.

In summary, the present data indicated that ASP enhanced both renal and hepatic EPO production via stabilizing HIF-2α protein and attenuating inflammatory suppression, restored EPOR signaling systems, and increased iron availability by suppressing hepcidin and inflammation, thereby correcting anemia in CKD rats (**Figure 9**). By stimulating endogenous EPO expression, ASP treatment may lead to a rise of plasma EPO within physiological ranges as opposed to high peaks observed with intravenous ESA therapy. And enhanced iron mobilization could reduce the use of intravenous iron supplementation and improve EPO efficacy. Therefore, ASP may potentially be an alternative and/or adjunctive therapy for patients suffered from anemia of CKD.

## Author Contributions

KW, JW, JX, and SG carried out experiments and analyzed data. KW and FZ participated in experiment design. FZ, JW, QL, and PC analyzd data and wrote the manuscript. ML and YZ contributed to the revision of paper.

## Conflict of Interest Statement

The authors declare that the research was conducted in the absence of any commercial or financial relationships that could be construed as a potential conflict of interest.

## Abbreviations

Bcl-2: B-cell lymphoma 2
BCL-xL: B-cell lymphoma-extra large
BMP: bone morphogenetic protein
DMT1: divalent metal transport 1
EPO: erythropoietin
EPOR: erythropoietin receptor
GATA2: GATA binding protein 2
IL-1β: interleukin 1β
IL-6: interleukin 6
JAK: Janus kinase
MAPK: mitogen-activated protein kinase
NF-κB: nuclear factor kappa-light-chain-enhancer of activated B cells
PBS: phosphate-buffered saline
PI3K: phosphatidylinositol 3-kinase
SMAD: son of mother against decapentaplegic
STAT: signal transducer and activator of transcription
TfR1: transferrin receptor 1
TNFα: tumor necrosis factor alpha
VEGFα: vascular endothelial growth factor α.
