# Supplementary material for: Correction of Anemia in Chronic Kidney Disease With Angelica sinensis Polysaccharide via Restoring EPO Production and Improving Iron Availability
Source: Front Pharmacol. 2018 Jul 31;9:803. doi: 10.3389/fphar.2018.00803 (PMC6079227; doi:10.3389/fphar.2018.00803)
Supplement: Supplementary file 1 [file Image_1.PDF]

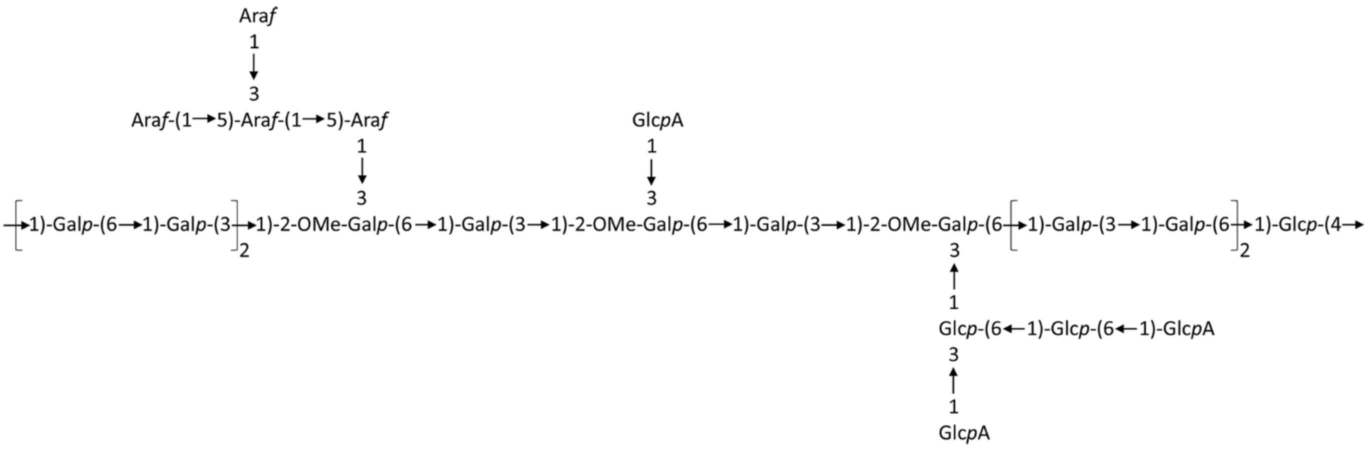

Supplemental Figure S1. Structure of the repeating unit of ASP. Ara*f*, arabinofuranose; Gal*p*, galactopyranose; Glc*p*A, glucopyranuronic acid; Glc*p*, glucopyranose.
