# Supplementary material for: Correction of Anemia in Chronic Kidney Disease With Angelica sinensis Polysaccharide via Restoring EPO Production and Improving Iron Availability
Source: Front Pharmacol. 2018 Jul 31;9:803. doi: 10.3389/fphar.2018.00803 (PMC6079227; doi:10.3389/fphar.2018.00803)
Supplement: Supplementary file 2 [file Image_2.pdf]

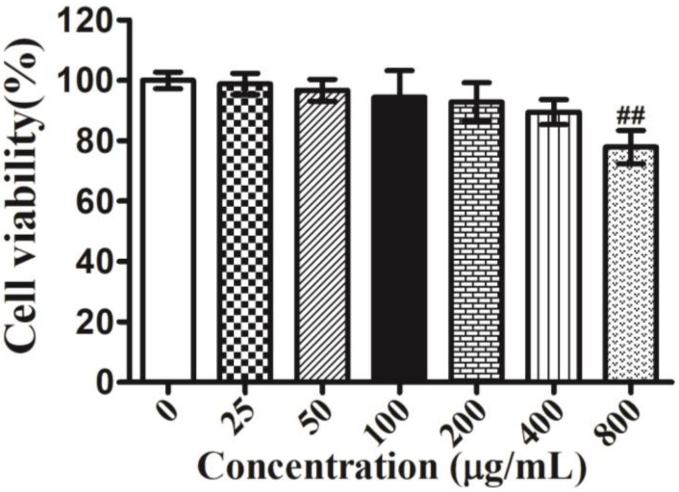

Supplemental Figure S2. Cytotoxicology of ASP in the Hep3B cell lines. The cytotoxicity test of ASP against Hep3B cells was performed by MTT assay. $^{##}$ $P < 0.01$ compared with the “0” group.
