# Supplementary material for: Correction of Anemia in Chronic Kidney Disease With Angelica sinensis Polysaccharide via Restoring EPO Production and Improving Iron Availability
Source: Front Pharmacol. 2018 Jul 31;9:803. doi: 10.3389/fphar.2018.00803 (PMC6079227; doi:10.3389/fphar.2018.00803)
Supplement: Supplementary file 3 [file Image_3.pdf]

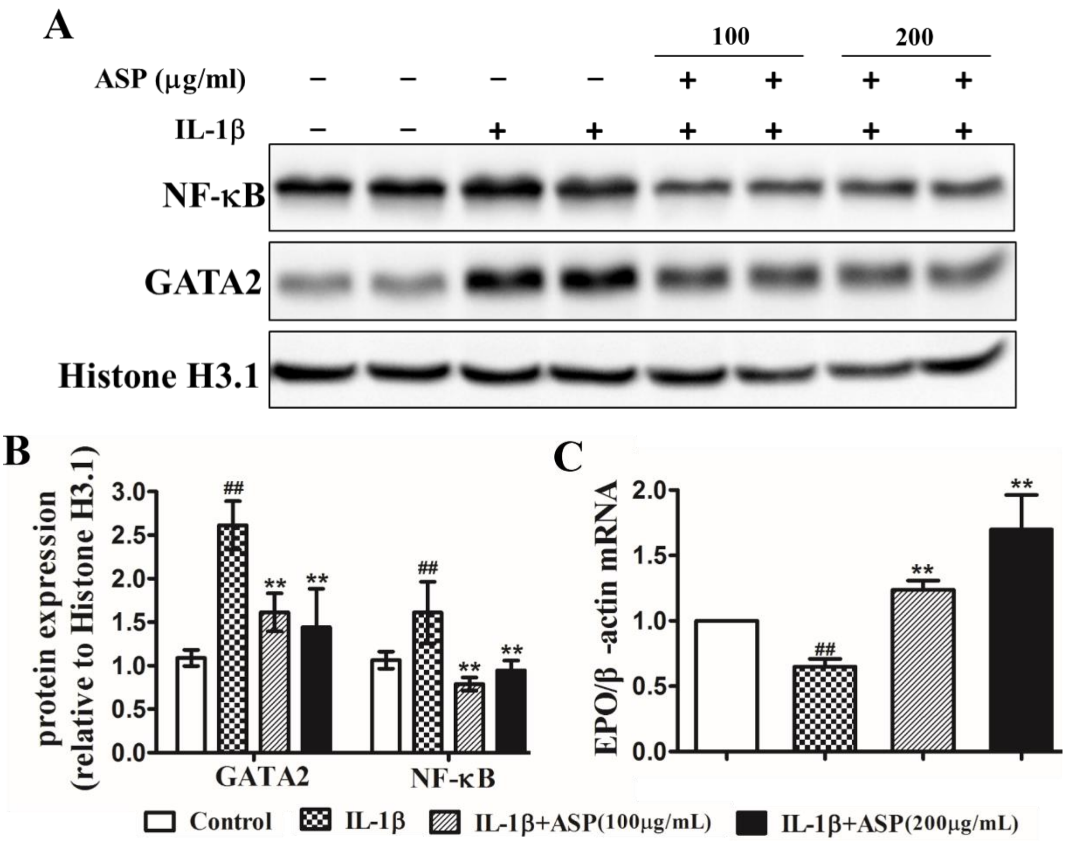

Supplemental Figure S3. IL-1β-induced activation of NF-κB and GATA2 and repression of EPO in Hep3B cells are prevented by ASP. (A, B and C) Hep3B cells were induced by 25μg/mL IL-1β in presence or absence of ASP (100 μg/mL, 200 μg/mL). (A and B) Nuclear GATA2 and NF-κB expression. (C) EPO mRNA expression. Histone H3.1 served as nuclear loading control. Values are means ± SEM from 3 independent experiments. $^{\#\#}$ $P < 0.01$ compared with the control group; * $P < 0.05$ and ** $P < 0.01$compared with the IL-1β group.
