# Supplementary material for: Correction of Anemia in Chronic Kidney Disease With Angelica sinensis Polysaccharide via Restoring EPO Production and Improving Iron Availability
Source: Front Pharmacol. 2018 Jul 31;9:803. doi: 10.3389/fphar.2018.00803 (PMC6079227; doi:10.3389/fphar.2018.00803)
Supplement: Supplementary file 4 [file Table_1.PDF]

Supplemental Table S1.

Sequences of the primers used in quantitative PCR reactions.

	Target gene	Forward (5′-3′)	Reverse (5′-3′)
Rat			
	TNF-α	GCCTCCCTCTCATCAGTTCTAT	TTTGCTACGACGTGGGCTA
	IL-1β	CTCTGTGACTCGTGGGATGATG	CACTTGTTGGCTTATGTTCTGTCC
	IL-6	CTGTCTCGAGCCCACCAGGAA	GGCTGGAAGTCTCTTGCGGA
	Hamp 1	CTGAGCAGCGGTGCCTATCT	TTGGTGTCTCGCTTCCTTCG
	DMT1	CAACTCTACCCTGGCTGTGG	GTCATGGTGGAGCTCTGTCC
	TfR1(TfRc)	GCTCGTGGAGACTACTTCCG	GCCCCAGAAGATGTGTCGG
	EPO	GAATGAAGGTGGAAGAACAGG	AGCACCCGAAGCAGTGAAGT
	PHD1	GCTGCTGCGTTGGTTAC	GCCTCCTGGTTCTCTTG
	PHD2	CTGGGACGCCAAGGTGA	CAATGTCAGCAAACTGG
	PHD3	GTTCAGCCCTCCTATGC	ACCACCGTCAGTCTTTA
	VEGFα	GAAGTTCATGGACGTCTACCAG	CATCTGCTATGCTGCAGGAAGCT
	EPOR	CTGGGAGGAAGCGGCGAACT	GCGGTGGTAGCGAGGAGAT
	Bcl-2	GGATGACTTCTCTCGTCGCTAC	TGACATCTCCCTGTTGACGCT
	Bax	CCAAGAAGCTGAGCGAGTGTCTC	AGTTGCCATCAGCAAACATGTCA
	Bcl-xL	GTAAACTGGGGTCGCATTGT	TGCTGCATTGTTCCCATAGA
	Fam132b	AGAAGAGGAGCAGGACCAAAG	TAGTGAGATCCCTGGTGCAGT
	β-actin	CACGATGGAGGGGCCGGACTCATC	TAAAGACCTCTATGCCAACACAGT
Human			
	EPO	TCATCTGTGACAGCCGAGTC	CAAGCTGCAGTGTTCAGCAC
	HIF-1α	ATCTCGGCGAAGTAAAGAATCTG	GTCACCATCATCTGTGAGAACC
	HIF-2α	CCAATCCAGCACCCATCCCAC	GTTGTAGATGACCGTCCCCTG
	β-actin	AGCGAGCATCCCCCAAAGTT	GGGCACGAAGGCTCATCATT
