# Supplementary material for: Correction of Anemia in Chronic Kidney Disease With Angelica sinensis Polysaccharide via Restoring EPO Production and Improving Iron Availability
Source: Front Pharmacol. 2018 Jul 31;9:803. doi: 10.3389/fphar.2018.00803 (PMC6079227; doi:10.3389/fphar.2018.00803)

Uncropped images of the original western blots

Correction of anemia in chronic kidney disease with *Angelica sinensis* polysaccharide via restoring EPO production and improving iron availability

Kaiping Wang [1], Jun Wu [1], Jingya Xu [1], Saisai Gu [1], Qiang Li [2], Peng Cao [2], Mingming Li [2], Yu Zhang [2], Fang Zeng [2*]

[1]*Hubei Key Laboratory of Nature Medicinal Chemistry and Resource Evaluation, Tongji Medical College of Pharmacy, Huazhong University of Science and Technology, Wuhan, China*

[2] *Department of Pharmacy, Union Hospital of Huazhong University of Science and Technology, Wuhan, China*

*Corresponding author at: Department of Pharmacy, Union Hospital of Huazhong University of Science and Technology, No. 1277, Jiefang Road, 430022, Wuhan, China

E-mail address: fangzengwhuh @163.com (Fang Zeng)

Fig. 2A

HIF-1α

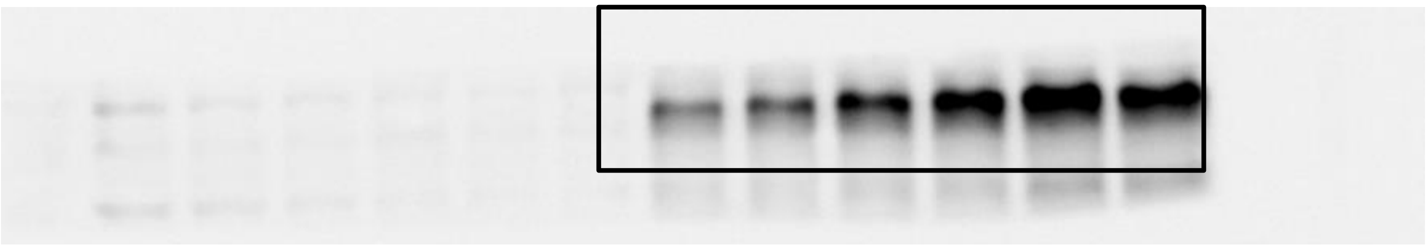

HIF-2α

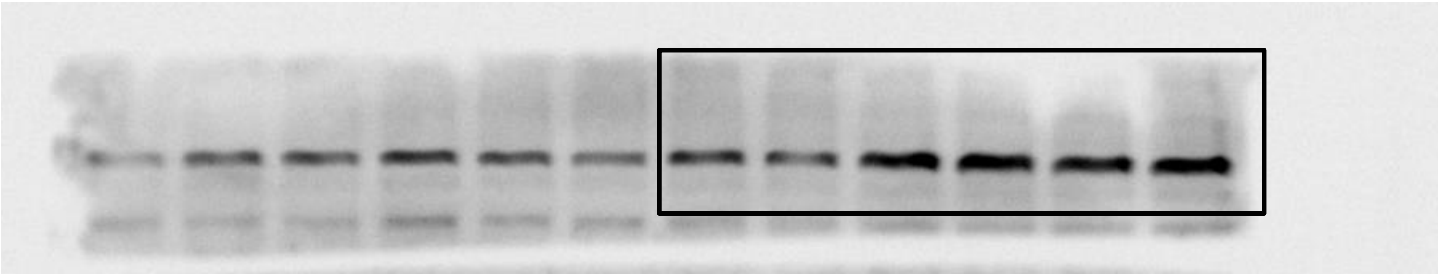

β-actin

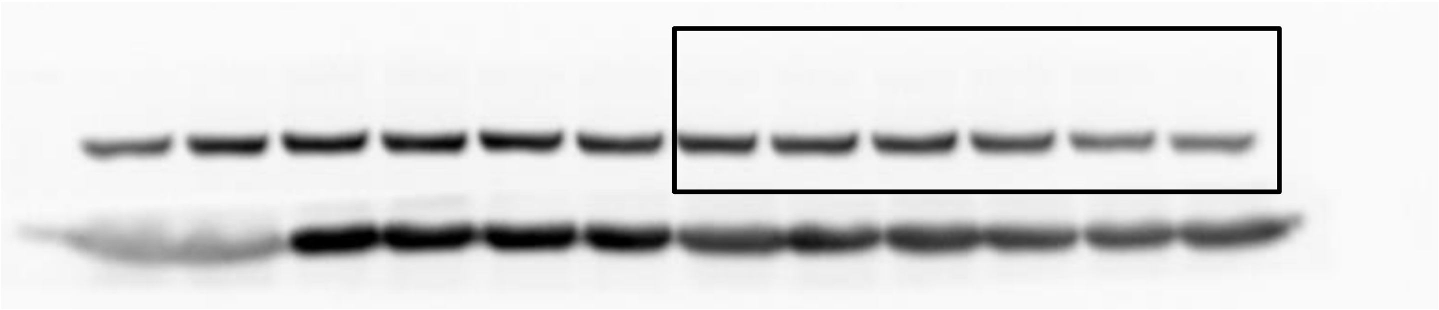

Fig. 2E

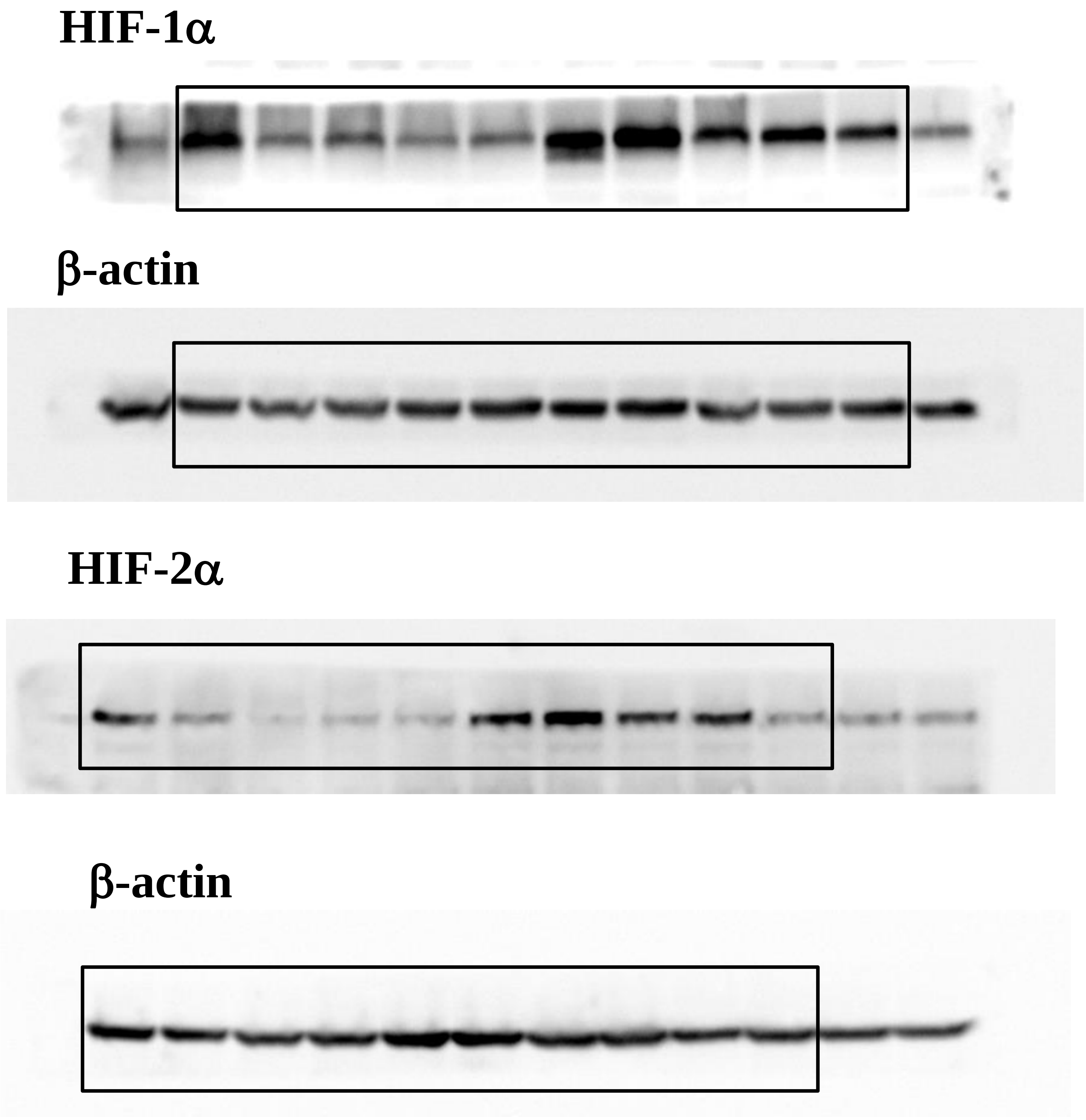

Fig. 3A

GATA2

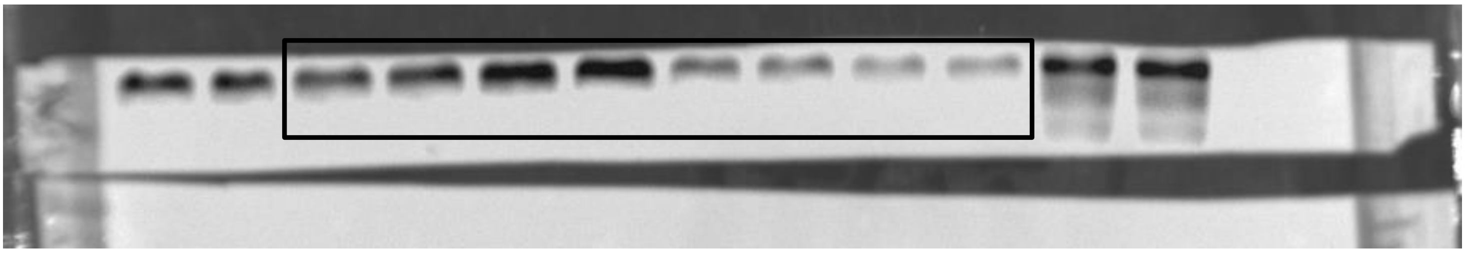

Histone H3.1

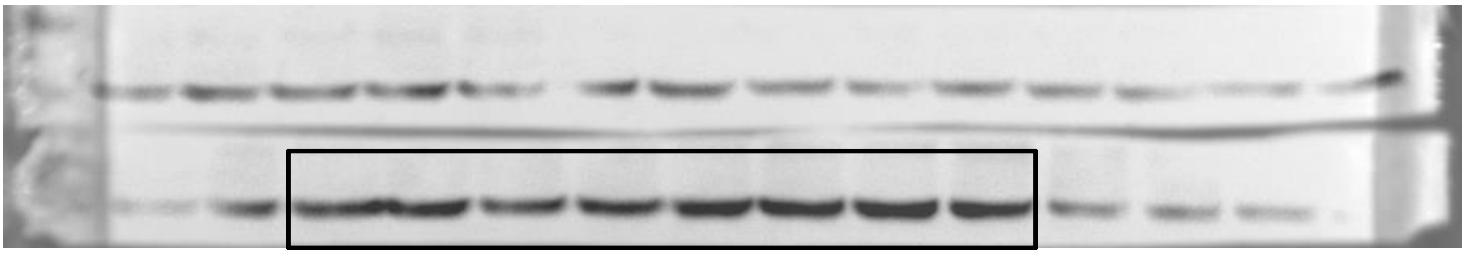

Fig. 3D

GATA2

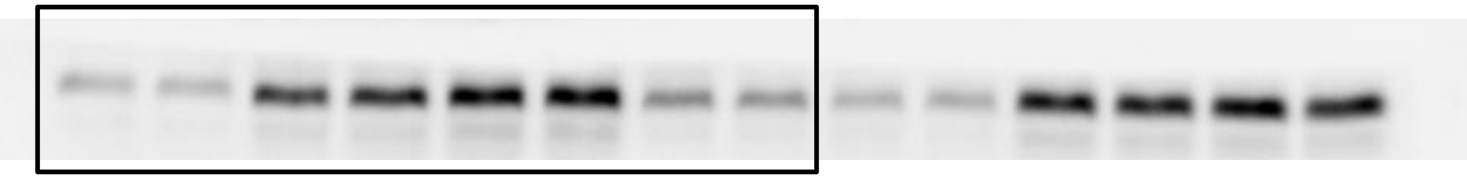

NF-κB

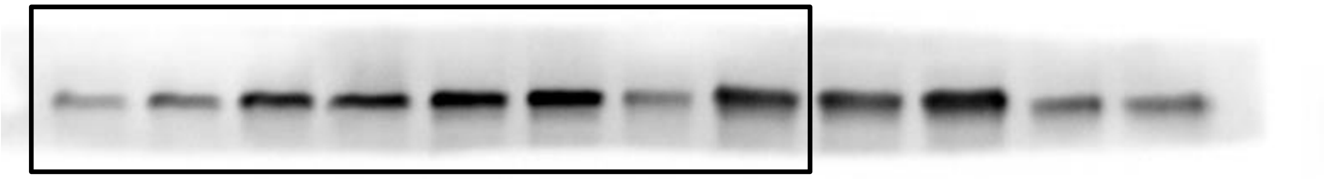

Histone H3.1

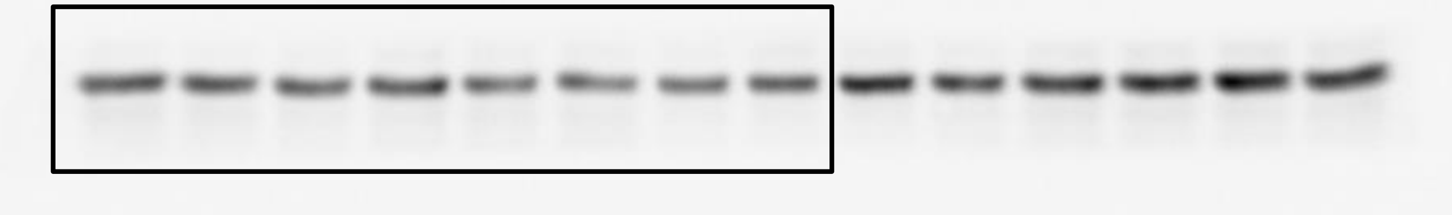

Fig 6.D

HIF-1α

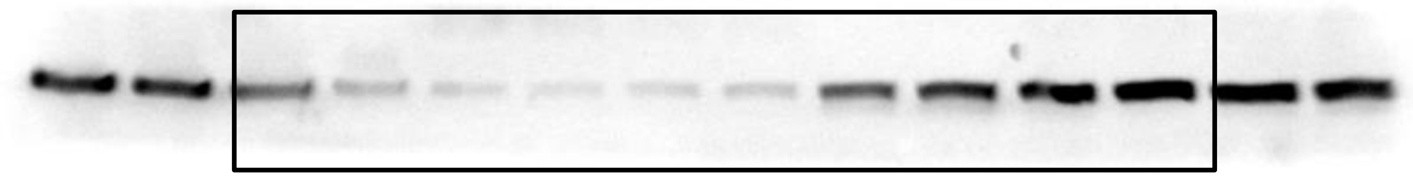

HIF-2α

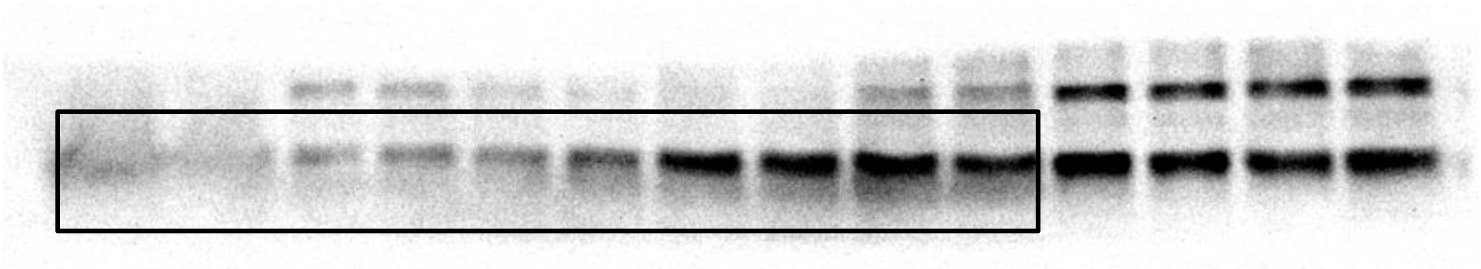

NF-κB

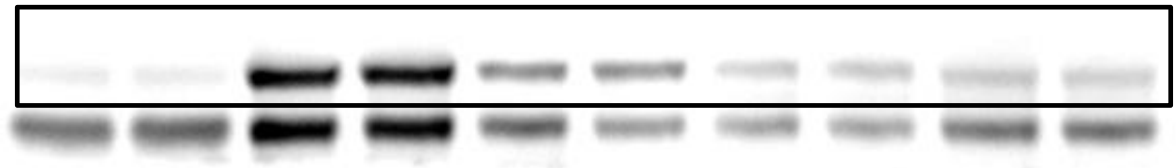

GATA2

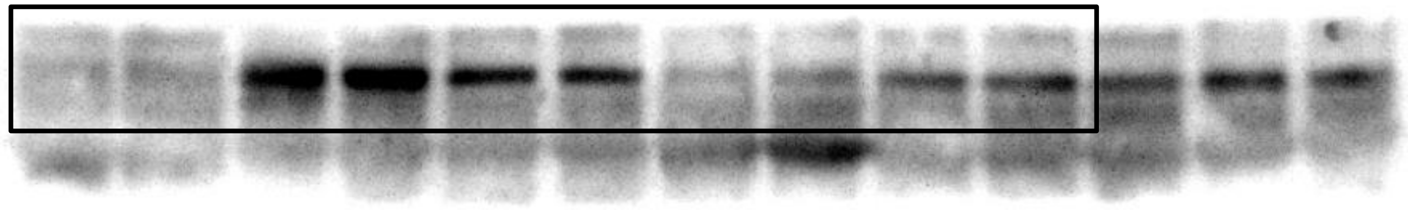

β-actin

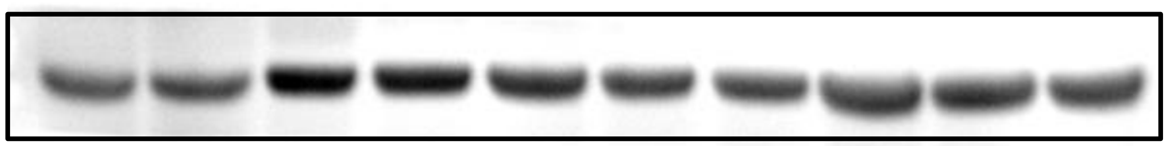

Fig 6.E

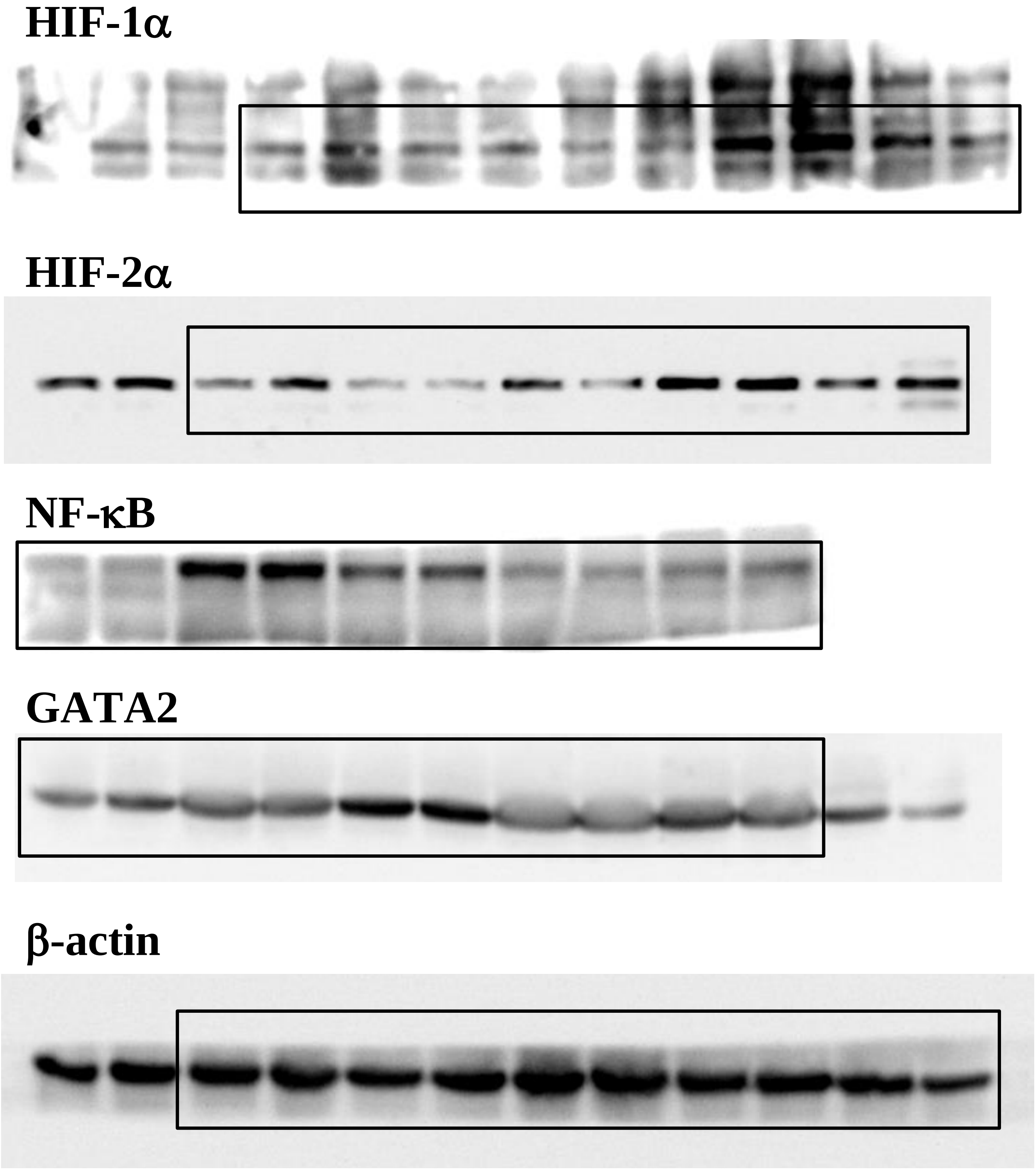

Fig. 7B

p-JAK2

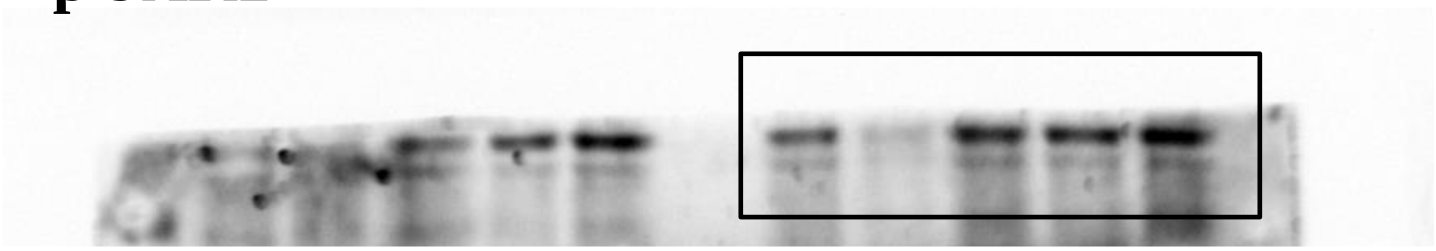

p-STAT5

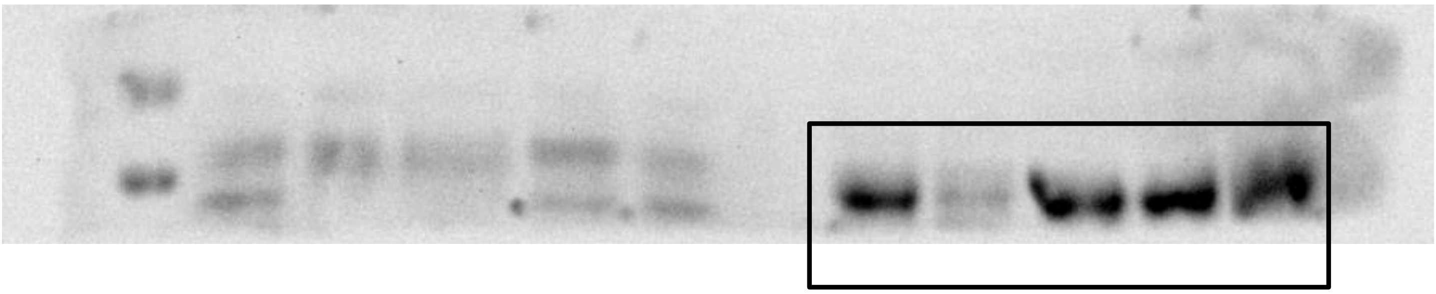

p-PI3K

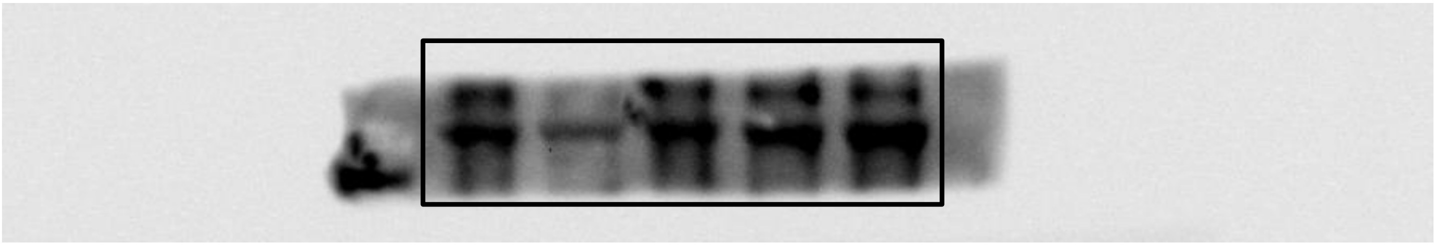

p-Akt

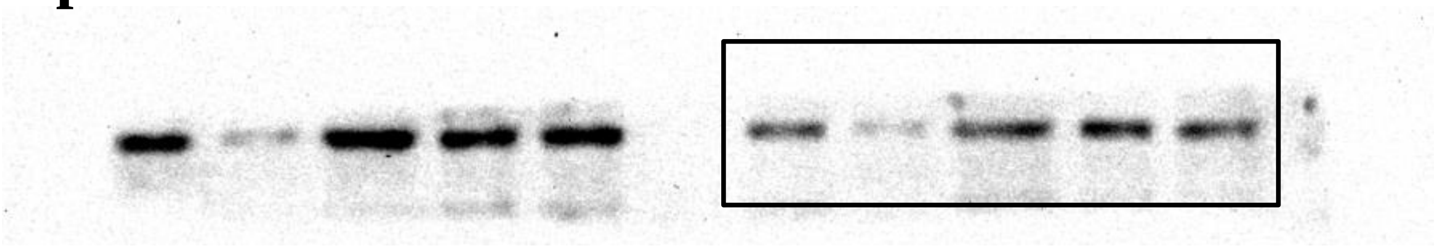

β-actin

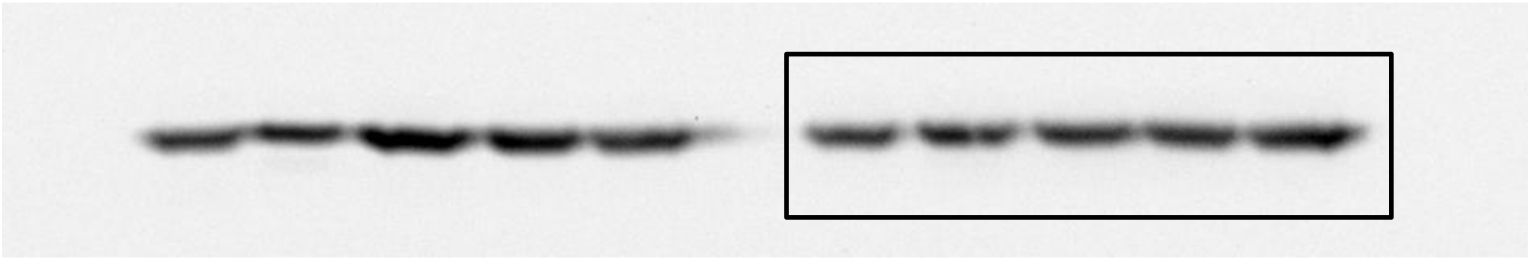

Fig 8.D

p-STAT3

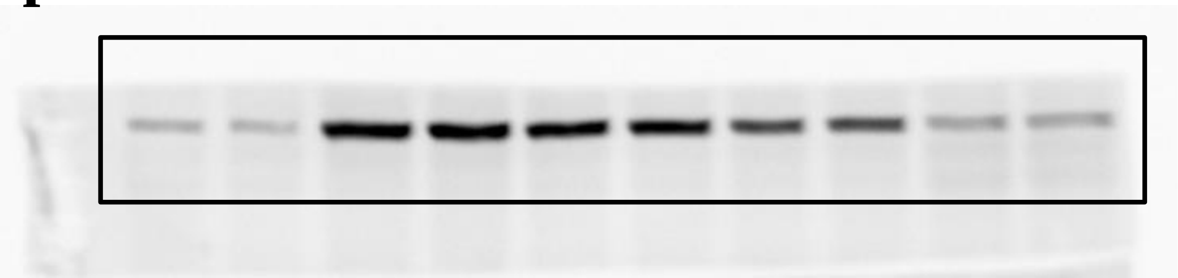

p-SMAD1/5/8

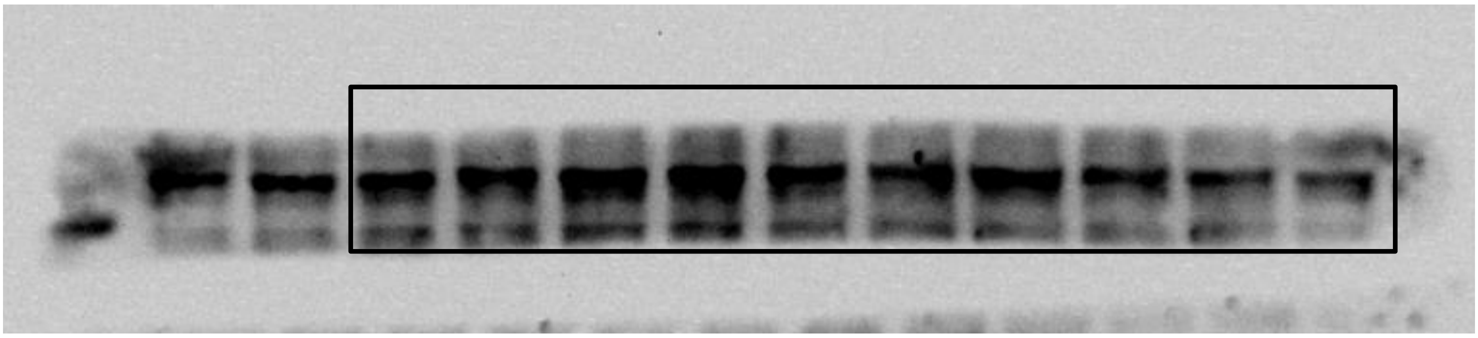

β-actin

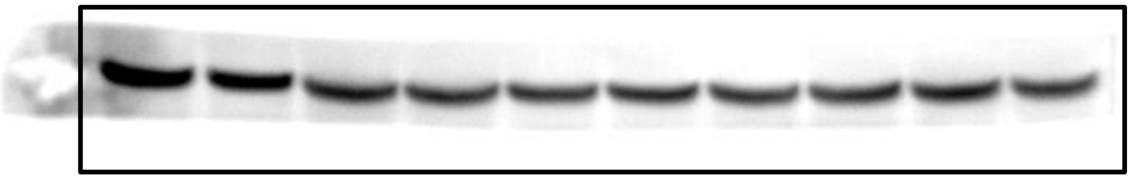

Fig 8.F

Spleen

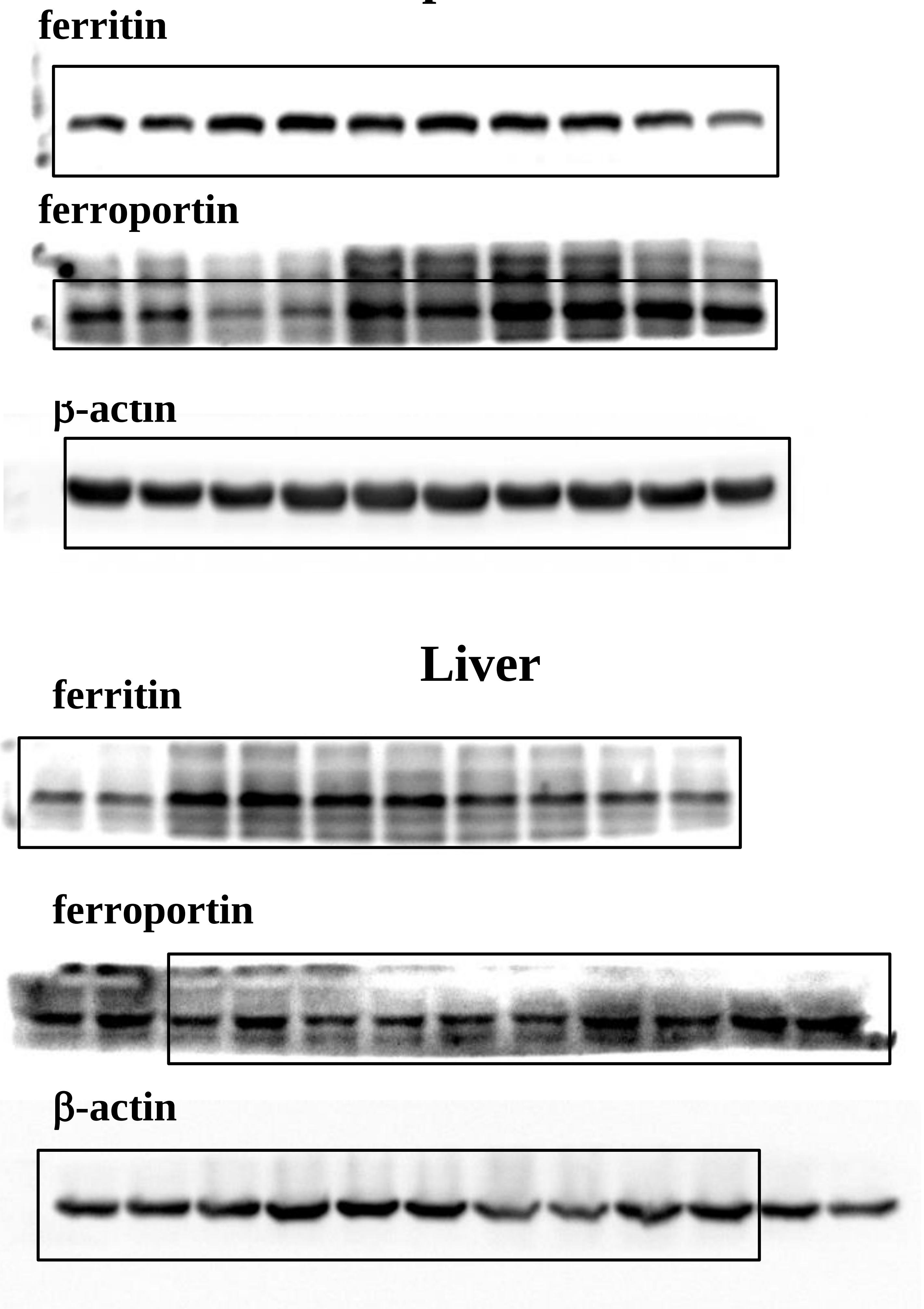

Supplemental Fig. S2

NF-κB

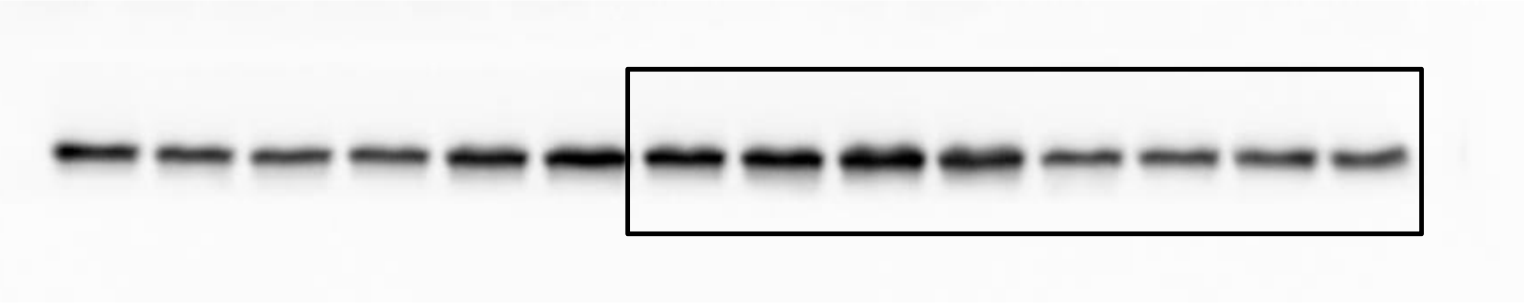

GATA2

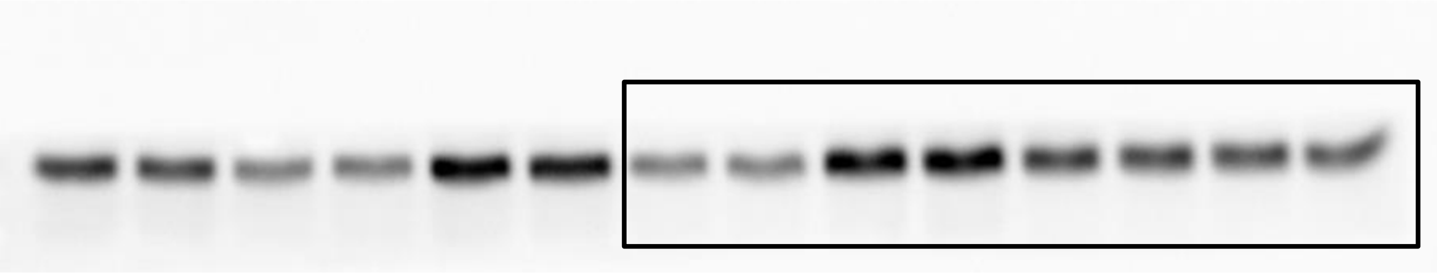

Histone H3.1

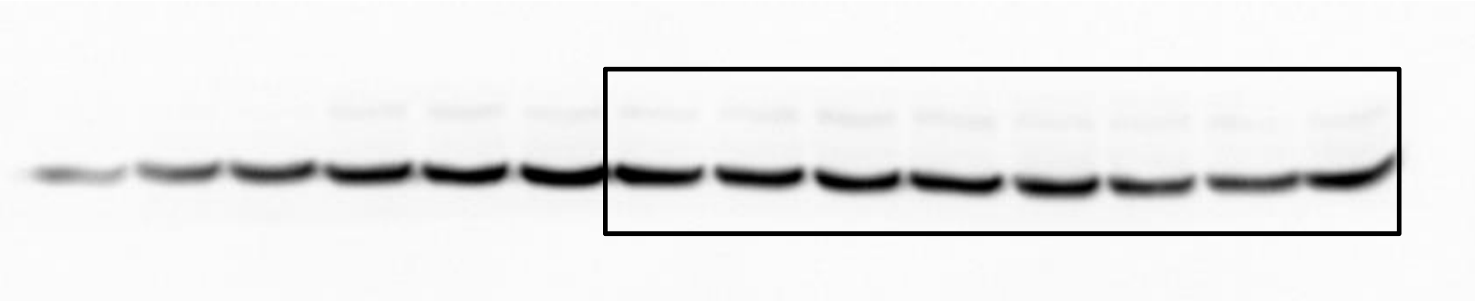

Fig. 2A

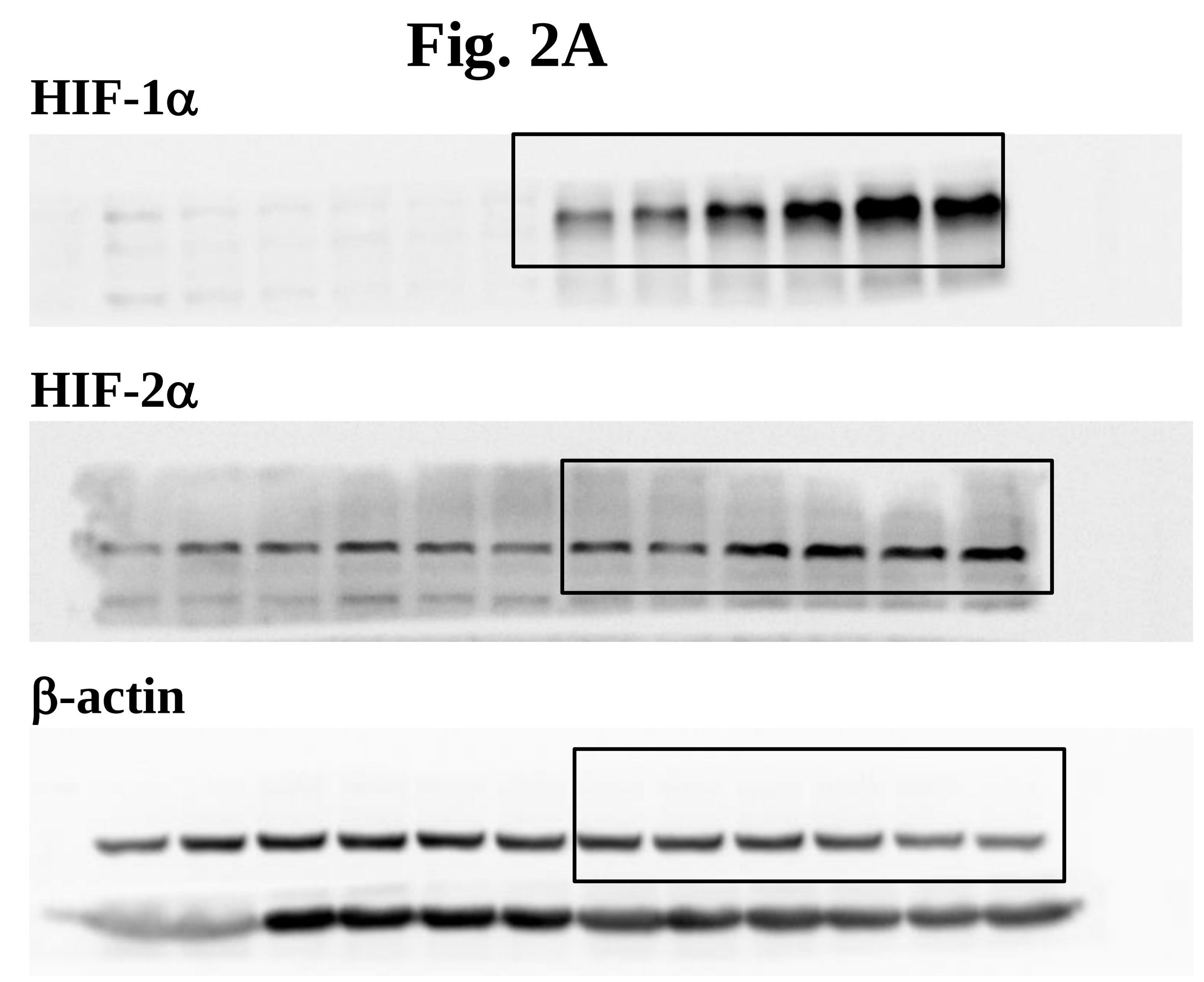

Fig. 2E

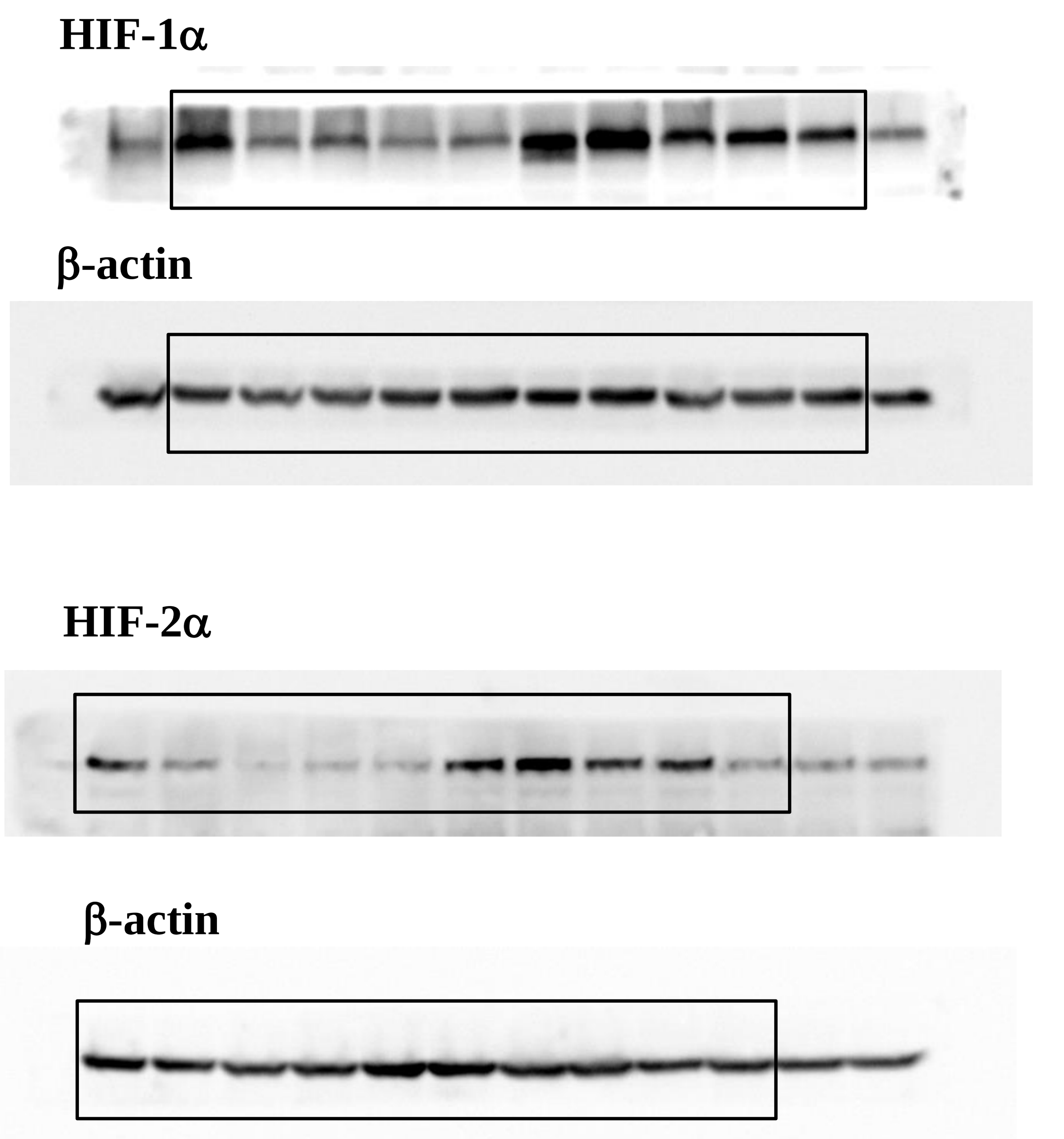

Fig. 3A

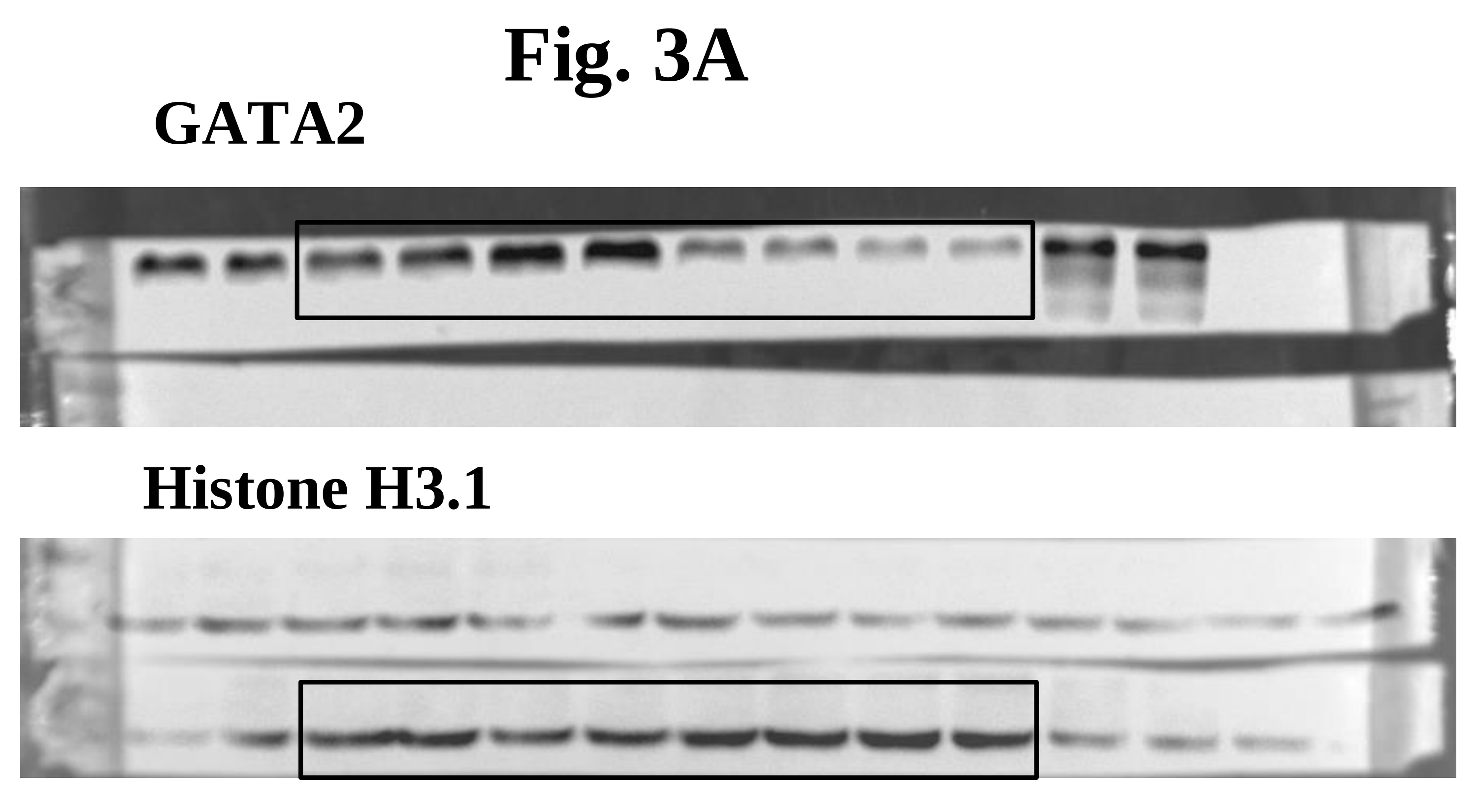

Fig. 3D

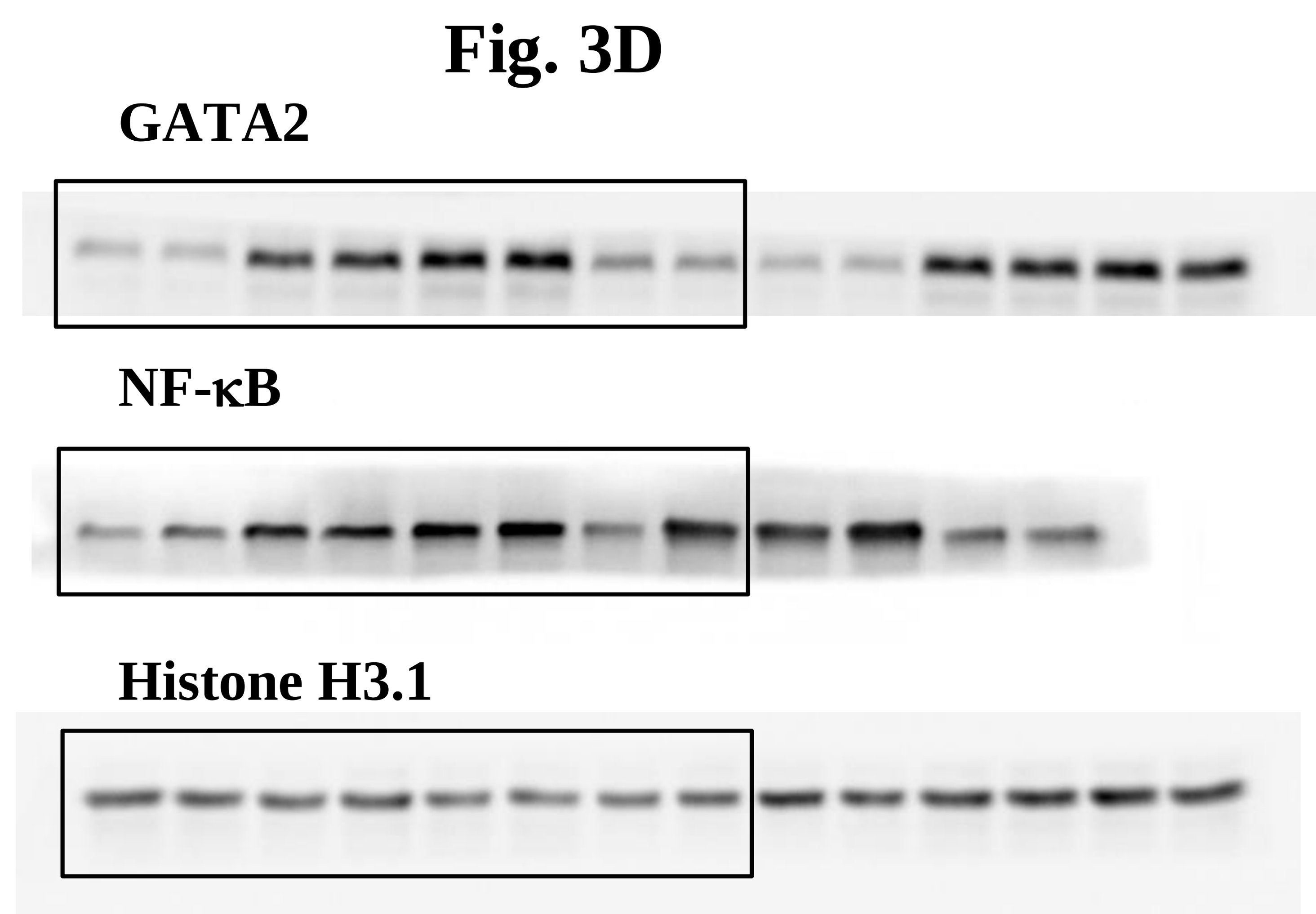

Fig 6.D

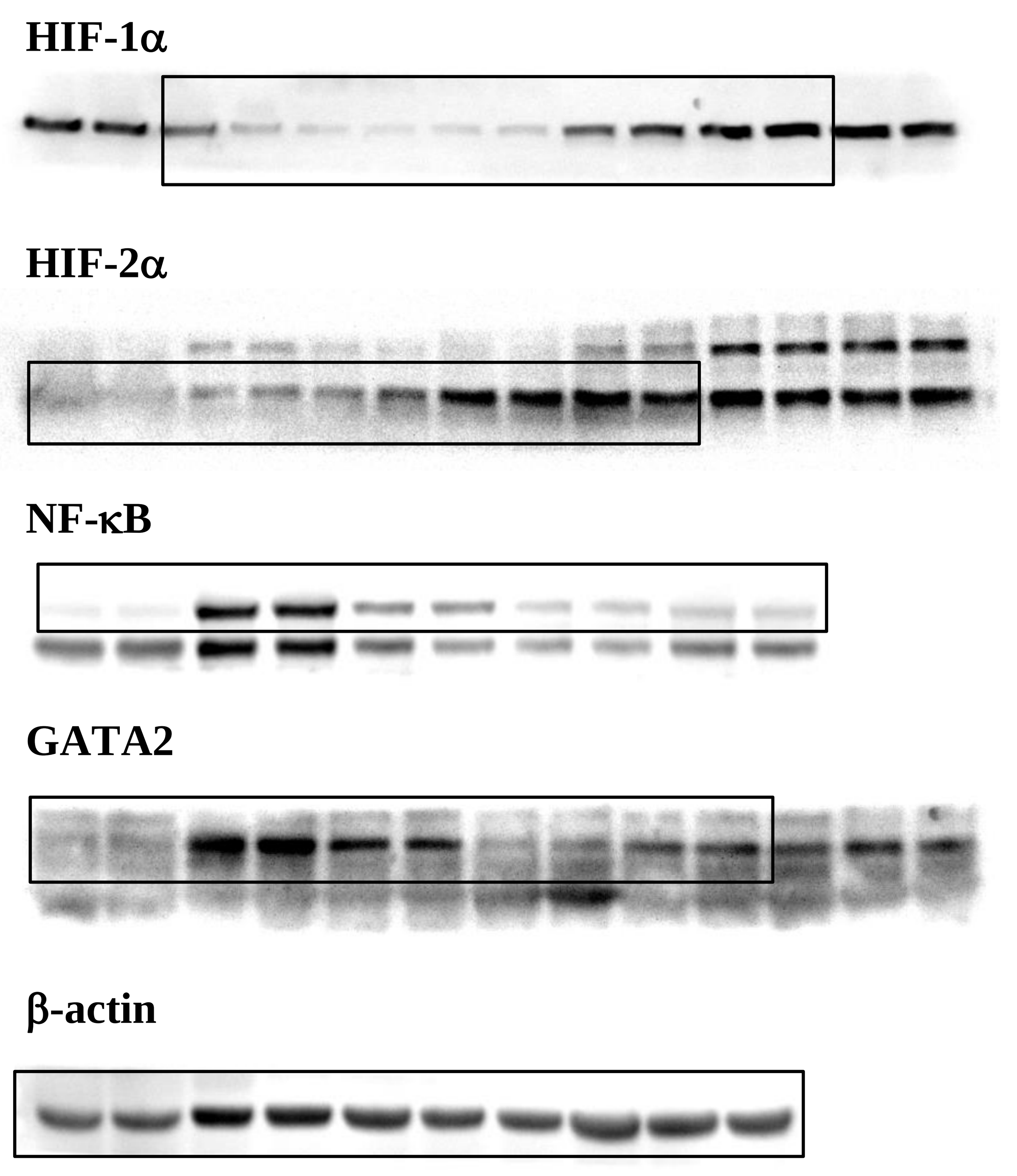

Fig 6.E

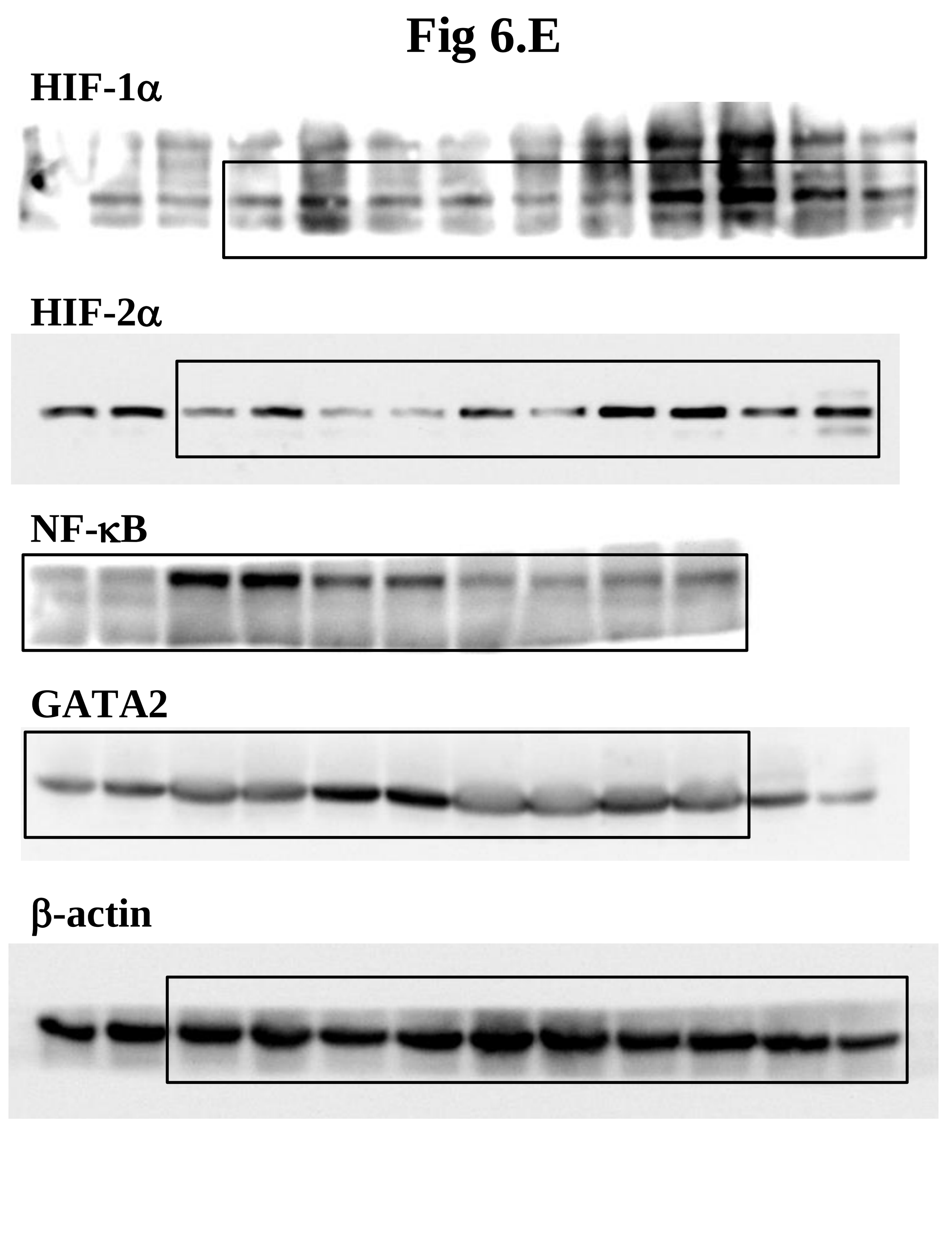

Fig. 7B

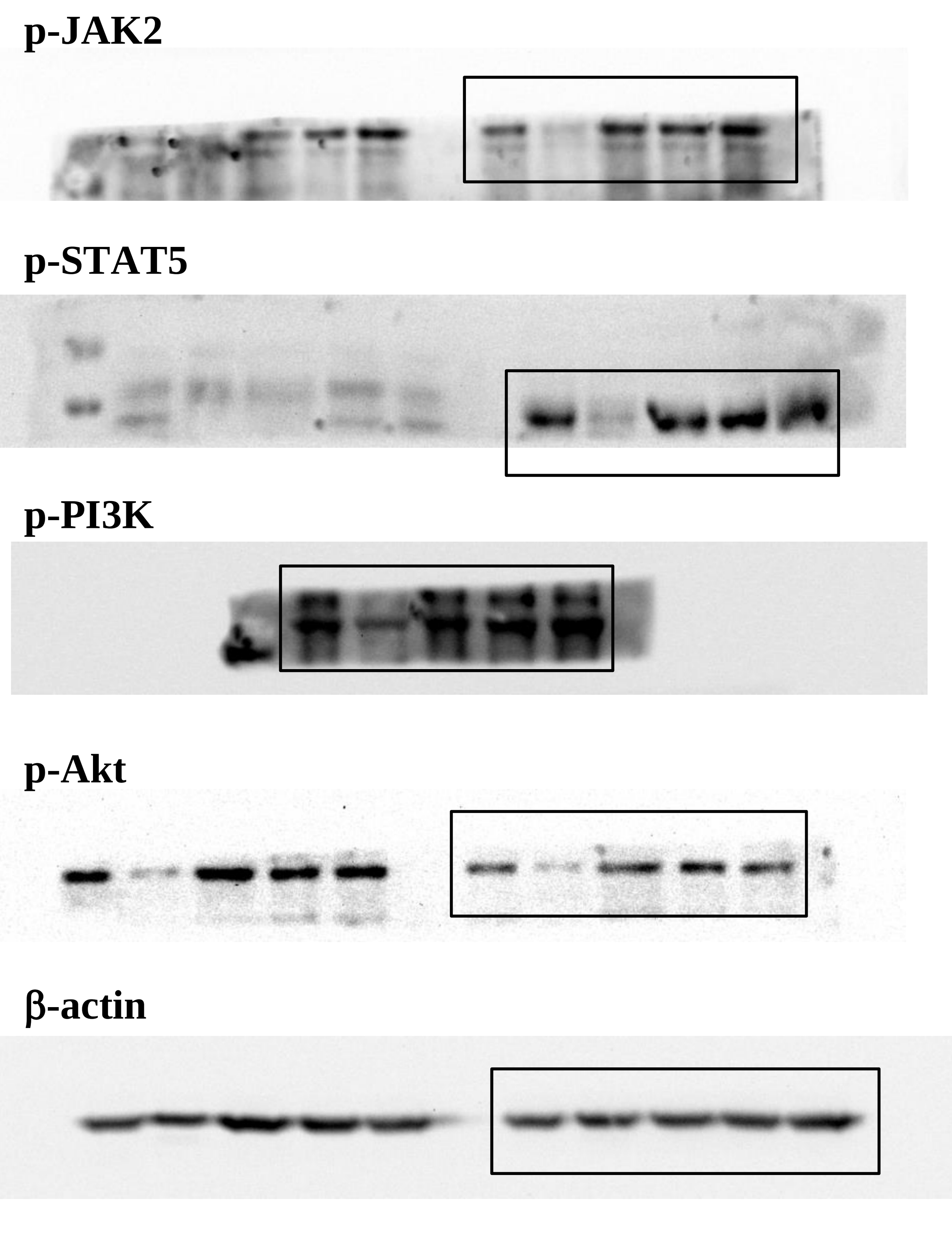

Fig 8.D

p-STAT3

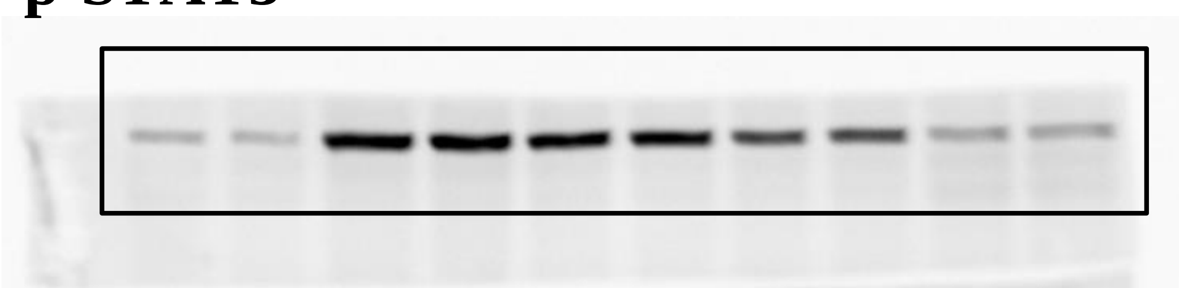

p-SMAD1/5/8

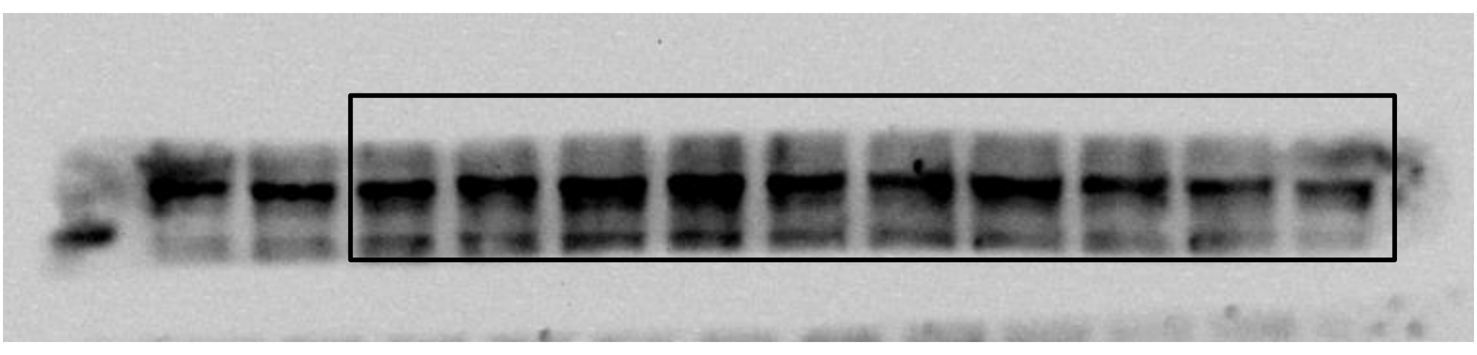

β-actin

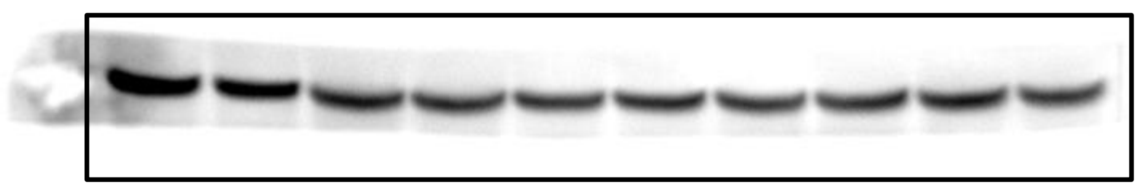

Fig 8.F
Spleen

ferritin

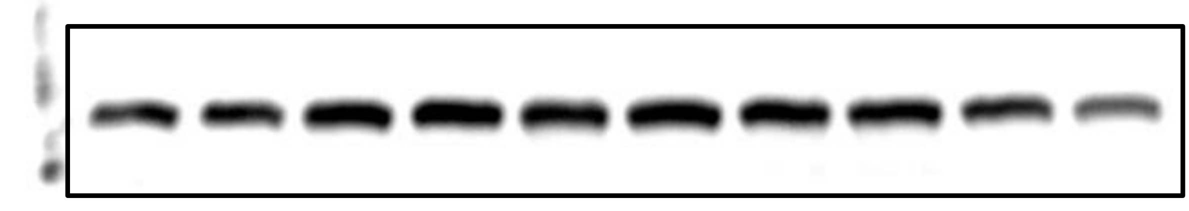

ferroportin

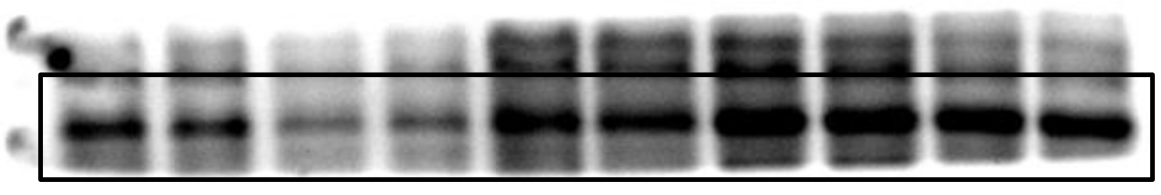

β-actin

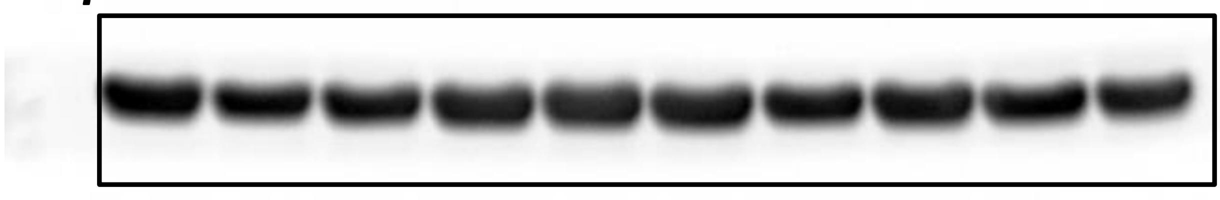

Liver

ferritin

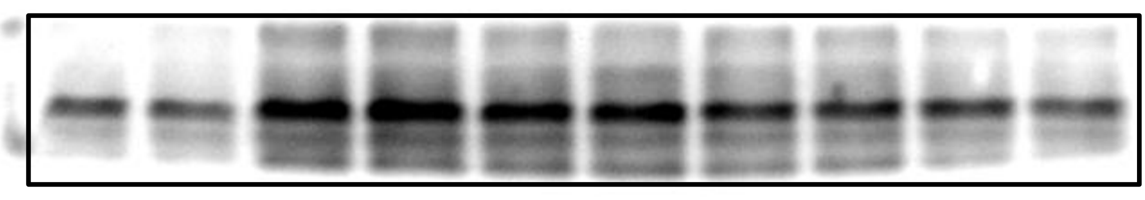

ferroportin

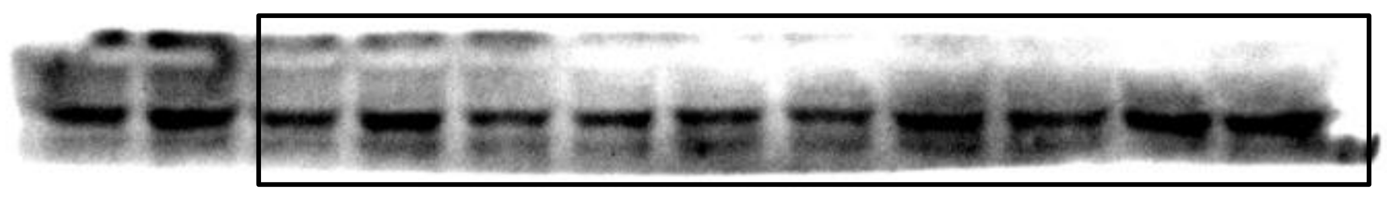

β-actin

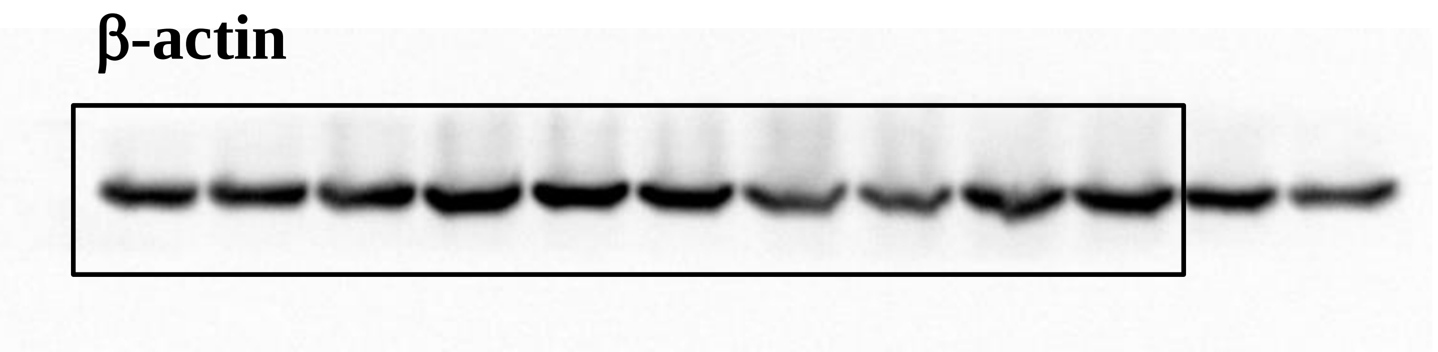

Supplemental Fig. S2

NF-κB

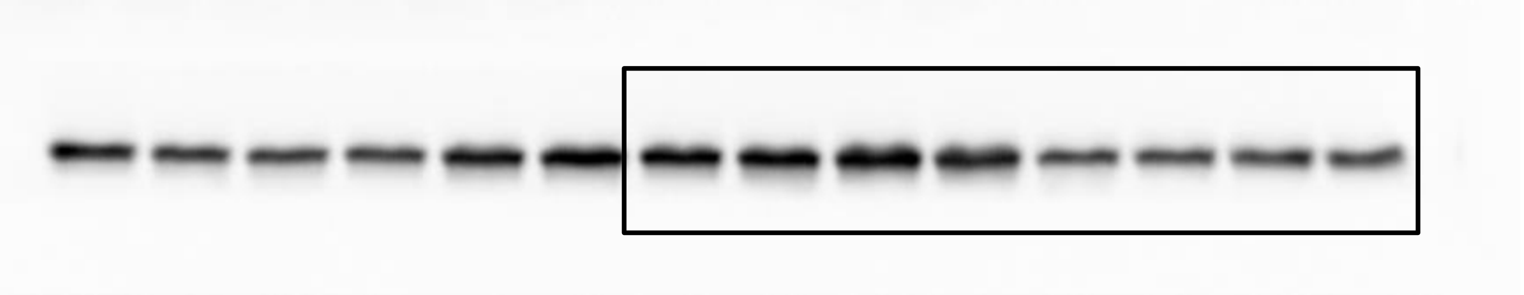

GATA2

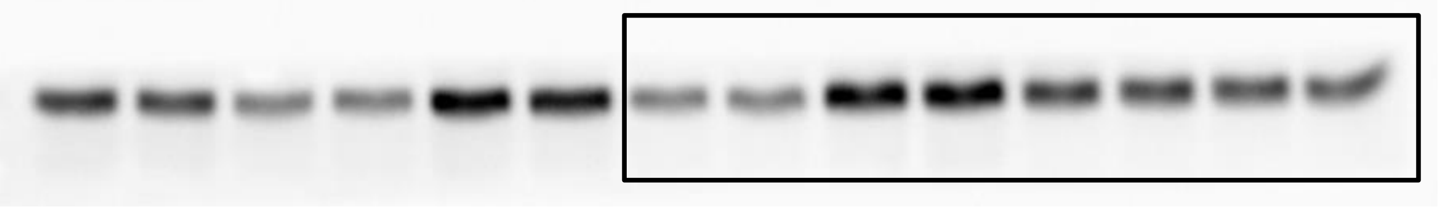

Histone H3.1

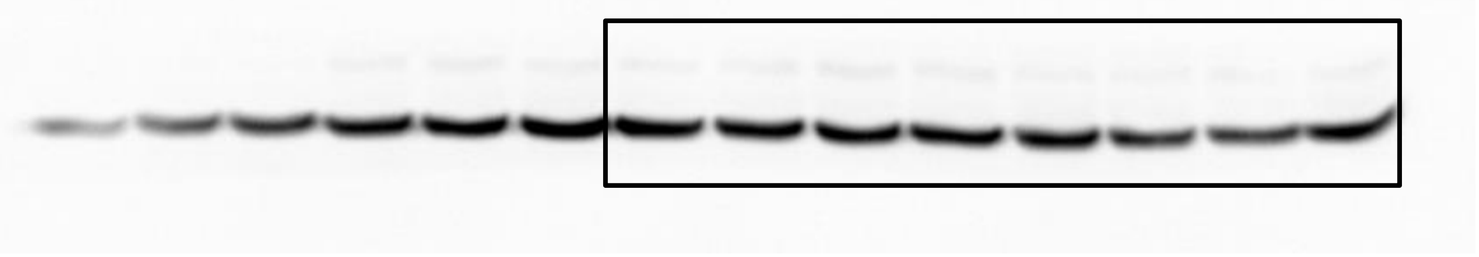

Supplement: Supplementary file 5 [file Data_Sheet_1.pdf]
